# Lead Exposure and Nutritional Status Among Bangladeshi Children: An Analysis Based on Bangladesh Multiple Indicator Cluster Survey

**DOI:** 10.3390/foods15213911

**Published:** 2026-10-31

**Authors:** Rafid Hassan, Ahadul Hassan Bhuiyan Konok, Tanjina Akter, Masum Ali, Md. Ruhul Amin, Sanjib Saha

**Affiliations:** 1 Department of Nutrition and Food Engineering, Daffodil International University, Dhaka 1216, Bangladesh; rafidinfs31@gmail.com; 2 Institute of Nutrition and Food Science, University of Dhaka, Dhaka 1000, Bangladesh; ahadulkonok@gmail.com (A.H.B.K.); ruhul.infs@du.ac.bd (M.R.A.); 3 International Centre for Diarrhoeal Disease Research, Bangladesh (icddr,b), Dhaka 1212, Bangladesh; tanjina2797@gmail.com; 4 Nutrition and Health Science, Laney Graduate School, Emory University, Atlanta, GA 30322, USA; mali54@emory.edu; 5 Department of Clinical Science (Malmo), Health Economics Unit, Lund University, 221 00 Lund, Sweden

**Keywords:** blood lead level, child undernutrition, composite index of anthropometric failure, stunting, wasting, underweight, heavy metal exposure, lead exposure, environmental health, Bangladesh, multiple indicator cluster survey

## Abstract

Bangladesh carries one of the world’s highest estimated childhood lead burdens alongside a substantial undernutrition burden, yet no nationally representative study has examined the association of blood lead levels (BLLs) with anthropometric failure. We analysed data from the 2025 Bangladesh Multiple Indicator Cluster Survey, including 10,495 children aged 12–59 months with BLLs and anthropometric measurements. Venous blood lead, arsenic, cadmium, and mercury were measured alongside anthropometry. Associations of BLLs with height-for-age (HAZ), weight-for-age (WAZ), and weight-for-height (WHZ) z-scores were estimated using linear regression; associations with stunting, wasting, underweight, and the Composite Index of Anthropometric Failure (CIAF, comprising six disaggregated failure categories) were estimated using modified Poisson regression yielding adjusted prevalence ratios (APRs). Models considered continuous standardised log-transformed BLLs and elevated BLLs (≥5 µg/dL), with and without adjustment for arsenic, cadmium, mercury, and covariates. The prevalence of stunting, wasting, underweight, and CIAF was 25.9%, 14.1%, 26.5%, and 38.2%, respectively. Elevated BLL (≥5 µg/dL) prevalence was 38.6%. Higher BLLs were significantly associated with lower HAZ (β = −0.075), WAZ (β = −0.062), and WHZ (β = −0.029) and with higher prevalence of stunting (APR = 1.09), wasting (APR = 1.08), underweight (APR = 1.08), and CIAF (APR = 1.07); estimates were similar after mutual adjustment for arsenic, cadmium, and mercury. Elevated BLLs (≥5 µg/dL) were not significantly associated with WHZ. BLLs were associated with several CIAF patterns, most strongly the combined stunting-wasting-underweight category (APR = 1.18), with significant associations present across every category involving stunting. In exploratory analyses, elevated BLLs were significantly associated with household soil lead concentration (APR = 1.22), occupational contact with lead-related work (APR = 1.08), and child pica behaviour (APR = 1.18), and were inversely associated with solid fuel use for cooking (APR = 0.78). Elevated BLL prevalence varied substantially by district (range 4.4–93.7%), with higher burden concentrated in the central–eastern region. BLLs were associated with impaired growth and specific patterns of anthropometric failure among Bangladeshi children. These findings support prioritising lead source control and integrating growth monitoring with environmental exposure surveillance in national child health programmes. Household soil lead, occupational exposure, and child pica behaviour represent modifiable targets for source-control interventions.

## 1. Introduction

Child undernutrition remains one of the most persistent global public health challenges. In 2024, an estimated 150.2 million children under five years of age were stunted and 42.8 million were wasted worldwide [1]. Undernutrition is a leading underlying contributor to child morbidity and mortality. Despite decades of global nutrition programming, progress toward the 2030 Sustainable Development Goal targets remains insufficient in most countries [1].

Alongside this nutritional burden, environmental lead exposure represents a pervasive and preventable threat to child health. Lead has no known beneficial biological function and can adversely affect the nervous, hematological, cardiovascular, renal, and immune systems. Children are particularly vulnerable because of their developing physiology and greater susceptibility to lead absorption, and no blood lead levels (BLLs) have been identified as safe: evidence of adverse neurodevelopmental effects extends to relatively low exposure levels [2]. Globally, approximately one in three children—up to 800 million—have been estimated to have BLLs ≥ 5 µg/dL, with a substantial proportion of affected children living in South Asia [3]. The consequences extend beyond individual health. Global modelling has estimated large losses in cognitive potential and substantial economic costs attributable to lead exposure (US$6.0 trillion globally in 2019), with a disproportionate share of this burden borne by low- and middle-income countries (LMICs) [4].

Bangladesh exhibits this dual burden at a national scale. In 2025, stunting, wasting, and underweight affected approximately 24%, 12.9%, and 23% of children under five years of age, respectively [5], and a substantial proportion of Bangladeshi children experience more than one form of anthropometric failure simultaneously in 2022 [6]. Bangladesh also carries one of the largest population-level burdens of childhood lead exposure documented globally. UNICEF previously estimated that approximately 35.5 million Bangladeshi children had BLLs ≥ 5 µg/dL, placing Bangladesh among the countries with the largest absolute numbers of lead-exposed children globally [3]. Around 38.4% of children aged 12–59 months had elevated BLLs (≥5 µg/dL), with a mean BLL of 5.21 µg/dL in 2025 [5]. This exposure has been attributed to dietary contamination, most notably lead-adulterated turmeric, alongside informal used lead-acid battery recycling, lead-containing paints, soldered food containers, and other occupational and environmental sources [3,7,8,9].

Both lead exposure and childhood undernutrition can have lasting consequences for physical growth and human capital across the life course, and a growing body of epidemiological evidence links childhood lead exposure to impaired physical growth. Existing Bangladeshi studies of lead and child growth, however, have relied on geographically selected samples, limiting the ability to yield nationally generalisable estimates [10,11]. Furthermore, previous research has primarily focused on single anthropometric indicators, rather than examining whether lead exposure is associated with multiple, co-occurring dimensions of growth impairment, such as those captured by the Composite Index of Anthropometric Failure (CIAF). The 2025 Bangladesh Multiple Indicator Cluster Survey (MICS), which incorporated venous blood lead measurement alongside standard anthropometry, provides a nationally representative opportunity to address this gap. Therefore, this study aimed to examine the associations of BLLs among Bangladeshi children aged 12–59 months with height-for-age, weight-for-age, and weight-for-height z-scores; the corresponding binary undernutrition indicators; and composite anthropometric failure as defined by the CIAF.

## 2. Materials and Methods

### 2.1. Study Design and Participants

This study was a secondary analysis of data from the 2025 Bangladesh MICS (7th round), a nationally representative household survey implemented by the Bangladesh Bureau of Statistics (BBS). MICS7 used a two-stage, stratified cluster sampling design based on a sampling frame from the 2022 Population and Housing Census. In the first stage, 3149 enumeration areas (EAs) were selected as primary sampling units using probability-based sampling. In the second stage, 20 households were systematically selected from each sampled EA, resulting in a total of 62,980 selected households. The sampling design comprised 131 sampling strata formed by urban and rural areas within the eight administrative divisions, the 64 districts, and Dhaka North, Dhaka South and Chattogram City Corporations. Details are provided elsewhere [5].

Venous blood specimens from children and household soil samples were collected through a country-specific module of the survey [5]. In each household with at least one age-eligible child, one child aged 12–59 months was selected for blood collection; where more than one was present, the Kish grid embedded in the computer-assisted personal interviewing (CAPI) application selected one child at random. A total of 18,913 children aged 12–59 months were identified in the sampled households, of whom 17,132 were eligible for blood collection after this one-child-per-household selection. A blood test result was recorded for 10,661 eligible children, and 10,634 had a valid laboratory result. After excluding children with implausible or flagged values or missing data on analysis variables, the analytic sample comprised 10,495 children (complete-case analysis; Supplementary Figure S1). The 166 children with a recorded blood result who were excluded (27 with an invalid laboratory result, 136 with implausible or flagged values and 3 with missing maternal education) were compared with the analytic sample (Supplementary Table S1).

### 2.2. Outcome Variables

Child anthropometry (height/length and weight) was converted to height-for-age (HAZ), weight-for-age (WAZ), and weight-for-height (WHZ) z-scores using the WHO Child Growth Standards, excluding biologically implausible values as per the WHO flagging algorithm [12]. Stunting, underweight, and wasting were defined as HAZ, WAZ, and WHZ below −2 SD, respectively [12]. The CIAF classified each child into one of seven mutually exclusive groups (no failure; wasting only; wasting and underweight; stunting, wasting, and underweight; stunting and underweight; stunting only; underweight only) [13]. CIAF was additionally collapsed into a binary indicator (any failure vs. no failure).

### 2.3. Exposure Variables

Venous blood specimens for heavy metal analysis were collected by trained personnel using aseptic precautions and sterile disposable equipment, then transported and analysed following the standardised biomarker collection and laboratory quality-assurance and quality-control protocol specified for the 2025 Bangladesh MICS7 biomarker module [5]. Blood lead, arsenic, cadmium and mercury concentrations were measured at the International Centre for Diarrhoeal Disease Research, Bangladesh (icddr,b), Dhaka, under a collaboration between the BBS, the Institute of Epidemiology, Disease Control and Research (IEDCR) and UNICEF [5]. BLLs (µg/dL) were natural log-transformed to reduce right-skewness and standardised to a z-score and analysed continuously as the primary exposure. BLLs were additionally dichotomised at ≥5 µg/dL as elevated BLLs, consistent with the MICS7 protocol threshold [5], and examined in parallel models. Blood arsenic, cadmium, and mercury concentrations (µg/L) were similarly log-transformed and standardised and were included in the mutually adjusted models as co-occurring exposures.

### 2.4. Covariates and Confounding Framework

Covariates were selected a priori based on the prior literature establishing pathways to both blood lead exposure and child undernutrition [5,6,14,15,16,17,18]. The primary model was adjusted for child sex, child age group in months (12–23, 24–35, 36–47, 48–59), maternal education (pre-primary/none, primary, secondary, higher), place of residence (rural, urban), administrative division (Barishal, Chattogram, Dhaka, Khulna, Mymensingh, Rajshahi, Rangpur, Sylhet), and household wealth quintile (poorest, poorer, middle, richer, richest).

### 2.5. Statistical Analysis

All analyses accounted for the complex survey design, including clustering within primary sampling units, stratification and the blood-specific sampling weight (chblweight). Statistical significance was defined as two-sided *p* < 0.05. Descriptive characteristics were summarised as unweighted frequencies and survey-weighted percentages, and bivariate associations between background characteristics and each anthropometric outcome were assessed using Rao–Scott design-based chi-square tests. Metal co-exposure was described by classifying each child according to the number of metals (0–4) with a concentration in the highest exposure category, defined as BLL ≥ 5 µg/dL for lead and the highest survey-weighted quartile of the distribution for arsenic, cadmium and mercury (Supplementary Table S2). Pairwise Spearman correlation coefficients were estimated among the four log-transformed metal concentrations (Supplementary Figure S2). Variance inflation factors (VIFs) were calculated for all exposures and covariates in the mutually adjusted models, indicating no substantial multicollinearity at VIF < 10.

Associations of BLLs with the three continuous outcomes (HAZ, WAZ and WHZ) were estimated using linear regression and associations with the binary outcomes (stunting, wasting, underweight and any CIAF) were estimated using modified Poisson regression with robust variance estimation, which yields adjusted prevalence ratios (PRs) and was selected because the outcomes were common [19,20,21]. Each of the six disaggregated CIAF categories was modelled in the same way as a separate binary outcome (category vs. no failure). For every outcome, two models were fitted: Model 1 included BLLs with the covariates described above, and Model 2 additionally included arsenic, cadmium and mercury as mutually adjusted co-occurring exposures. Coefficients represent the adjusted mean difference in z-score, or the adjusted PR, per 1-SD increase in log-transformed concentration, and the same two-model structure was applied with elevated BLLs (≥5 µg/dL) as the exposure. The associations of arsenic, cadmium and mercury with the seven growth outcomes were also estimated in single-pollutant and mutually adjusted models (Supplementary Table S3). To examine whether the continuous and threshold-based findings reflected a non-linear dose–response relationship, ln BLLs were additionally modelled using a restricted cubic spline, and a step change or a change in slope at 5 µg/dL was tested, both with and without adjustment for the other three metals (Supplementary Table S4 and Figures S3 and S4; details are given in the table footnote).

As a supplementary analysis to contextualise Bangladesh’s national BLLs burden, household, environmental, and demographic correlates of elevated BLLs were examined using modified Poisson regression. Candidate determinants included household soil lead concentration (quartile), neighbourhood informal lead-related industry proximity, household occupational contact with lead-related work, paint condition, child pica behaviour, cooking-fuel type, and sanitation access, together with the demographic and socioeconomic covariates described above; variable definitions and categorisation are provided in Supplementary Table S5. District-level prevalence of elevated BLLs, mean BLLs, and each growth-failure indicator (stunting, wasting, underweight, CIAF) were mapped spatially after combining each city corporation with its parent district (64 districts; Supplementary Table S6). Selection into the analytic sample was examined in a sensitivity analysis that re-weighted the analysed children by the inverse of their estimated probability of inclusion among the blood-eligible children (Supplementary Tables S7 and S8). Analyses were conducted in R (version 4.6.1) using the survey package.

## 3. Results

### 3.1. Participant Characteristics

Among 10,634 children aged 12–59 months with a valid blood metal laboratory result, 10,495 (98.7%) had complete data and were included in the analytic sample. The prevalence of elevated BLLs (≥5 µg/dL) was 38.6%. Over half of the children were male (52.5%), and the largest proportion were aged 48–59 months (28.2%). More than half of mothers had completed secondary education (54.6%). The sample was predominantly rural (70.7%), with the largest proportions of children in Dhaka (24.1%) and Chattogram (23.2%) divisions (Table 1).

Among the four metals, 27.8% of children were not in the highest exposure category for any metal, 39.8% were in this category for one metal, 24.2% for two, 7.2% for three and 0.9% for all four (Supplementary Table S2). Spearman correlations among the four metals were weak to modest; the strongest correlation was observed between lead and cadmium (ρ = 0.23), while correlations among the remaining metal pairs ranged from −0.03 to 0.13 (Figure S2).

### 3.2. Household, Environmental, and Demographic Determinants of Elevated BLLs

Children in the highest quartile of household soil lead concentration had a significantly higher adjusted prevalence of elevated BLLs than those in the lowest quartile (APR = 1.22, 95% CI: 1.11–1.33, *p* < 0.001), with a corresponding dose–response relationship across quartiles. Household occupational contact with lead-related work was associated with a modestly higher prevalence of elevated BLLs (APR = 1.08, 95% CI: 1.00–1.17, *p* = 0.049), and children with the most frequent pica behaviour had a higher prevalence than those with none (APR = 1.18, 95% CI: 1.05–1.33, *p* = 0.006). Solid fuel use for cooking was associated with a lower prevalence of elevated BLLs (APR = 0.78, 95% CI: 0.72–0.85, *p* < 0.001). Several demographic and socioeconomic covariates were also independently associated with elevated BLLs in the adjusted model: male sex (APR = 1.15, 95% CI: 1.09–1.22, *p* < 0.001), younger child age (APR = 1.18, 95% CI: 1.08–1.28, *p* < 0.001), lower maternal education (APR = 1.47, 95% CI: 1.30–1.66, *p* < 0.001), urban residence (APR = 1.08, 95% CI: 1.00–1.17, *p* = 0.045), and administrative division, with Chattogram, Dhaka and Sylhet divisions showing a higher prevalence, and Khulna, Rajshahi and Rangpur divisions a lower prevalence, than Barishal (all *p* < 0.001) (Supplementary Table S9).

### 3.3. Distribution of Undernutrition

Overall, the prevalence of stunting, wasting, underweight, and CIAF was 25.9%, 14.1%, 26.5%, and 38.2%, respectively (Table 2). Elevated BLLs were associated with a higher prevalence of stunting (27.5% vs. 24.8%, *p* = 0.014) but not with wasting, underweight, or CIAF prevalence in bivariate analysis. Stunting prevalence decreased with increasing child age (*p* < 0.001), whereas wasting (*p* = 0.018) and underweight (*p* = 0.034) increased. Maternal education and household wealth showed strong inverse gradients with all four undernutrition indicators. Undernutrition prevalence also varied significantly by division, ranging from 21.9% (Dhaka) to 40.0% (Sylhet) for stunting, 11.7% (Dhaka) to 17.3% (Rajshahi) for wasting, 19.8% (Dhaka) to 35.4% (Sylhet) for underweight, and 32.4% (Dhaka) to 50.9% (Sylhet) for CIAF.

### 3.4. Geographic Distribution of Elevated BLLs and Anthropometric Failure at District Level

**Figure 1:**
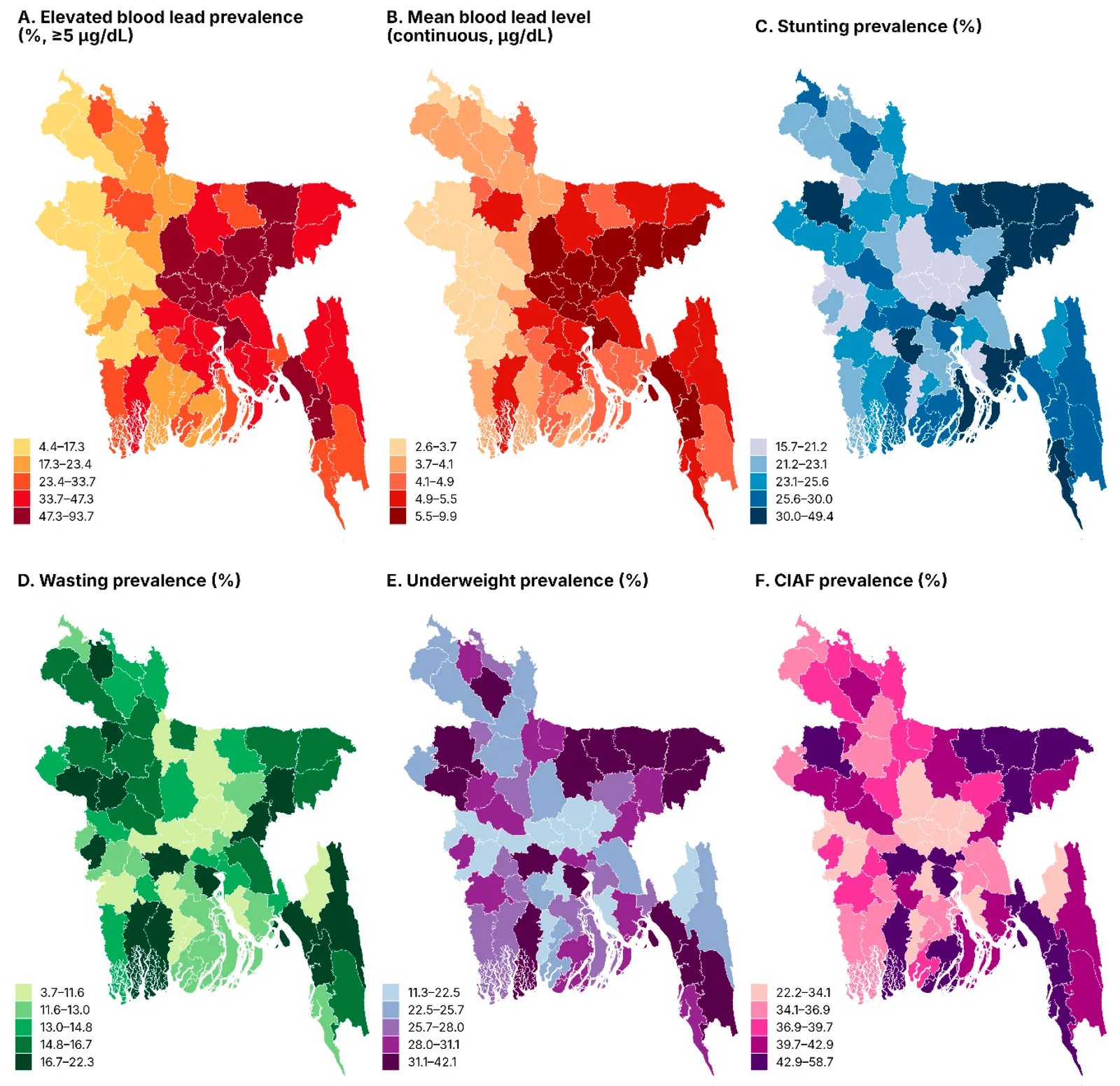
District-level prevalence of elevated BLLs, continuous BLLs, and child growth-failure indicators in Bangladesh MICS 2025 (children 12–59 months).

District-level elevated-BLLs prevalence ranged from 4.4% to 93.7%, with the highest burden concentrated in the central–eastern region. Stunting prevalence was higher in the north-eastern region, whereas wasting and underweight were more prevalent in peripheral districts (Figure 1; Supplementary Table S6). Two districts, Manikganj and Tangail, had fewer than 100 sampled children, so their estimates need cautious interpretation.

### 3.5. Associations of BLLs with Continuous Anthropometric Outcomes

Higher BLLs were significantly associated with lower HAZ (β = −0.075, 95% CI: −0.103, −0.046, *p* < 0.001), WAZ (β = −0.062, 95% CI: −0.088, −0.036, *p* < 0.001), and WHZ (β = −0.029, 95% CI: −0.055, −0.003, *p* = 0.031). The lead coefficients changed little between the single-pollutant and mutually adjusted models for all three outcomes (Table 3), consistent with the weak correlations among the four metals. The associations of arsenic, cadmium and mercury with the same outcomes are reported in Supplementary Table S3. Mercury and cadmium were not significantly associated with any outcome, whereas arsenic was associated with higher WAZ and WHZ and with a lower prevalence of underweight. Furthermore, children with BLLs ≥ 5 µg/dL had significantly lower HAZ (β = −0.10, 95% CI: −0.17, −0.04, *p* = 0.001) and WAZ (β = −0.07, 95% CI: −0.13, −0.01, *p* = 0.015) than children below this threshold. The association with WHZ was not statistically significant (Supplementary Table S10).

### 3.6. Associations of BLLs with Binary Anthropometric Outcomes

In the modified Poisson regression models, higher BLLs were associated with a significantly higher prevalence of stunting (APR = 1.09, 95% CI: 1.05, 1.14, *p* < 0.001), wasting (APR = 1.08, 95% CI: 1.02, 1.15, *p* = 0.012), underweight (APR = 1.08, 95% CI: 1.04, 1.13, *p* < 0.001), and CIAF (APR = 1.07, 95% CI: 1.04, 1.11, *p* < 0.001). These estimates changed minimally after mutual adjustment for arsenic, cadmium and mercury (Table 4). Furthermore, children with elevated BLLs were associated with a significantly higher prevalence of stunting (APR = 1.13, 95% CI: 1.04, 1.23, *p* = 0.003), underweight (APR = 1.11, 95% CI: 1.01, 1.21, *p* = 0.023), and CIAF (APR = 1.10, 95% CI: 1.03, 1.17, *p*= 0.004), but were not significantly associated with wasting (Supplementary Table S11).

### 3.7. Associations of BLLs with Composite Anthropometric Failure (CIAF)

Higher BLLs were associated with several CIAF patterns, including wasting with underweight (APR = 1.14, 95% CI: 1.04–1.25, *p* = 0.008), stunting with wasting and underweight (APR = 1.18, 95% CI: 1.06–1.31, *p* = 0.003), stunting with underweight (APR = 1.11, 95% CI: 1.04–1.19, *p* = 0.002), and stunting alone (APR = 1.12, 95% CI: 1.03–1.21, *p* = 0.007). These estimates changed minimally after adjustment for co-occurring metals (Table 5). Elevated BLLs were significantly associated with several CIAF patterns, including wasting with underweight (APR = 1.24, 95% CI: 1.02–1.51, *p* = 0.031), stunting with underweight (APR = 1.17, 95% CI: 1.02–1.33, *p* = 0.024), and stunting alone (APR = 1.22, 95% CI: 1.05–1.42, *p* = 0.010) (Supplementary Table S12).

## 4. Discussion

This study examined the associations of BLLs with linear and ponderal growth, and CIAF among Bangladeshi children aged 12–59 months. In this nationally representative sample, elevated BLLs (≥5 µg/dL) affected 38.6% of children. Higher BLLs were independently and consistently associated with anthropometric failure, including lower HAZ, WAZ, and WHZ, and a higher prevalence of stunting, wasting, underweight, and specific CIAF patterns. These associations persisted after adjustment for co-exposure to arsenic, cadmium, and mercury.

The prevalence of elevated BLLs observed here is lower than that reported in earlier Bangladeshi studies that sampled convenience groups or known contamination hotspots. In a multi-site study of children in Dhaka and Dinajpur, 54% had BLLs > 10 µg/dL [16]. In a Dhaka slum, 86.6% of children under two years had BLLs ≥ 5 µg/dL [11]. A rural birth cohort reported a prevalence at 20–40 months of age of 79% at one site and 14% at another [10]. Comparable, or higher, BLLs have been reported elsewhere in South Asia. A comprehensive review of BLLs across India, Bangladesh, Pakistan, Nepal, and Sri Lanka found that reported BLLs frequently exceeded the current US Centers for Disease Control and Prevention reference value of 3.5 µg/dL across the region [22]. A systematic review and meta-analysis of Indian children estimated a considerably higher pooled mean BLLs of 10.4 µg/dL, rising to 14.3 µg/dL among children with known lead-exposure sources [23]. Together, these findings position Bangladesh’s national BLL burden as substantial within South Asia, reflecting a shared, region-wide exposure problem.

Several overlapping pathways plausibly explain this high prevalence despite growing policy attention in Bangladesh [24,25]. Dietary contamination has been particularly well documented. Lead-chromate adulteration of turmeric, added to improve its colour and weight, was identified as a major source of childhood lead exposure in rural Bangladesh [7,9]. Other studies have implicated soldered food containers and metal cookware as additional dietary-contact sources [7,8]. Encouragingly, a coordinated food-safety enforcement effort combining rapid on-site lead detection with market-level regulatory action reduced detectable lead in turmeric from 47% to virtually none at Bangladesh’s largest wholesale market between 2019 and 2021 [26]. This effort was accompanied by a corresponding 30% decline in the BLLs of exposed mill workers [26], demonstrating that source-specific intervention can meaningfully reduce population exposure. Environmental sources have proved harder to control. Informal recycling of used lead-acid batteries (ULAB) is widespread in Bangladesh and generates severely contaminated soil around recycling sites, with soil lead concentrations exceeding 100,000 mg/kg documented near one abandoned site. Children’s BLLs fell substantially after soil remediation [27,28]. Consistent with this evidence, household soil lead concentration, occupational contact with lead-related work, and child pica behaviour were each independently associated with elevated BLLs in the present national sample, extending the informal-recycling and soil-ingestion pathways described above directly into the home. Beyond these point sources, broader environmental pollution from vehicle traffic, industrial emissions, and lead-containing paints and consumer products contributes to ambient and household-level exposure [8].

Younger children had a higher prevalence of elevated BLLs, which is consistent with their greater hand-to-mouth activity and floor contact in contaminated homes and yards and with the very high BLLs already reported among Bangladeshi children under three years of age [10,11]. Higher BLLs among boys have also been reported in another low- and middle-income setting [29], possibly reflecting more time outdoors in contact with soil and dust. Lower maternal education may be a marker of limited access to information on lead sources and of reduced capacity to avoid them, such as adulterated spices [9]. All of these explanations remain hypotheses, and future studies are required to examine them. Solid fuel use for cooking was, conversely, associated with a lower adjusted prevalence of elevated BLLs, which is a counterintuitive finding. The association was largely explained by geography: it attenuated markedly after adjustment for division (APR from 0.53 to 0.73) and further after adjustment for district (APR = 0.91; Supplementary Tables S13 and S14), consistent with solid-fuel use being less common in the highest-burden division, Dhaka (51.0% vs. 74–94% elsewhere), and more common in lower-burden regions. The small remaining association may reflect residual local-level confounding or chance, as this was one of several exploratory determinants examined. We therefore interpret it as regional confounding and not as a protective effect of cooking fuel; solid fuel use itself carries other health risks, notably household air pollution.

In this study, BLLs were inversely associated with HAZ, and elevated BLLs were associated with significantly higher stunting prevalence. This is consistent with two previous Bangladeshi studies. A two-site birth cohort of 618 children found that concurrent BLLs were associated with stunting at 20–40 months of age [10], and a cross-sectional study of children under two years in a Dhaka slum reported a similar association [11]. Comparable South Asian evidence beyond Bangladesh is limited. The regional review [22] and the Indian meta-analysis [23] discussed above both confirm widespread elevated exposure but do not directly model growth outcomes, indicating a gap in South Asian evidence linking BLLs to anthropometric failure outside Bangladesh. Evidence from other LMICs is broadly consistent. In Benin, children in the highest lead-exposure quartile had higher odds of stunting among girls in sex-specific analyses [30]. In Uganda, concurrent BLLs were inversely associated with HAZ among children aged 6–59 months [31]. Associations between lead exposure and impaired linear growth have also been reported among children in the Peruvian Amazon [32], Mexico [33], and Indonesia [34].

In this study, BLLs modelled continuously were associated with lower WHZ and a higher prevalence of wasting, whereas these associations were not apparent when lead was dichotomised at ≥5 µg/dL, as also reported for a threshold definition in the Dhaka slum cohort [11]. Direct comparison with other settings is limited: to our knowledge, no other Bangladeshi or South Asian study has modelled lead continuously against WHZ, and a Benin cohort found no overall association between lead-exposure quartiles and WHZ or wasting [30]. The difference between the two coding may partly reflect how exposure was modelled, because a single cut-point compares two broad groups and discards variation in lead within each, which can reduce the power to detect a small, graded association. Because both associations were modest and close to the significance level (*p* = 0.025 for WHZ and *p* = 0.025 for wasting), chance variation in the dichotomised analysis cannot be excluded as a contributor. Flexible modelling was consistent with a gradual relationship. In restricted cubic spline models, which impose no cut-point, we found no evidence of departure from linearity for WHZ (*p* = 0.323) or wasting (*p* = 0.315), or for the other five outcomes (all *p* for non-linearity >0.10). Direct tests for a step change or a change in slope at 5 µg/dL were also null for every outcome (all *p* ≥ 0.19), and the results were unchanged after adjustment for arsenic, cadmium and mercury (Supplementary Table S4 and Figures S3 and S4). These findings are therefore consistent with a continuous gradient and give no evidence of a threshold at 5 µg/dL. However, the WHZ association was small, the tests have limited power to detect modest curvature or a small break, and no correction was made for testing seven outcomes, so the results should be interpreted cautiously and replicated in other large populations.

Furthermore, this study found that the lead–WAZ association remained significant and stable across model specifications, with a corresponding increase in underweight prevalence. Among Bangladeshi children, this corroborates a previous cross-sectional study reporting associations between lead exposure and lower body mass index or underweight in a Dhaka slum [11]. No other nationally representative or South Asian study has examined this association directly. Evidence from other LMICs and developed-country settings assessing lead exposure alongside anthropometric or weight-related indicators has been mixed [30,32,35,36,37,38]. Differences in study populations, children’s ages, exposure levels, nutritional conditions, and analytical approaches may partly explain these inconsistent findings.

In addition, we also found that higher BLLs were also associated with several patterns of CIAF. The association was strongest for the most complex failure pattern (stunting, wasting, and underweight combined; APR = 1.18) and was present across every CIAF category that included a stunting component. To our knowledge, no prior study—in Bangladesh, South Asia, or elsewhere—has examined the association between BLLs, or any heavy metal, and CIAF specifically. Prior applications of CIAF in Bangladesh have characterised its association with sociodemographic determinants [6] and with infectious exposures. Asymptomatic Shigella infection, for example, was significantly associated with the CIAF in a Bangladeshi birth cohort [39], and the present findings extend this precedent to an environmental exposure. This pattern was consistent with the single-indicator findings. Every CIAF category reaching statistical significance included a stunting component, while isolated wasting and isolated underweight, without concurrent stunting, were both null. This corroborates the continuous-outcome findings, in which lead’s association was strongest for HAZ and WAZ and weakest for WHZ. Together, these results suggest that lead’s principal anthropometric signature in this population may operate through impaired linear growth. Given the cross-sectional design, however, these findings should be interpreted cautiously.

The association between lead exposure and different forms of undernutrition observed here can plausibly be explained through several biological pathways. First, lead has been shown to suppress growth hormone release and reduce circulating insulin-like growth factor-1 (IGF-1) in both children and animal models [40,41,42]. This could impair osteoblast function and bone mineralisation [43], providing biological plausibility for the observed effect on linear growth. A meta-analysis of prenatal lead exposure and birth weight found a significant inverse association [44], and low birth weight is an established risk factor for later stunting, offering one plausible early-life pathway. Second, lead exposure has been associated with gastrointestinal disturbance and reduced food intake in experimental and observational studies [45,46,47]. This pathway may be particularly relevant in Bangladesh, given the high background prevalence of environmental enteric dysfunction among young children. A recent Bangladeshi cohort found biomarkers of intestinal inflammation elevated in over 90% of infants, with poorer ponderal growth among children with greater intestinal inflammation [48]. Concurrent gut dysfunction could therefore compound any appetite-suppressing effect of lead on ponderal growth. Third, the relationship between lead exposure and nutritional status may be bidirectional. Iron deficiency can increase gastrointestinal lead absorption [49,50], and iron and other micronutrient deficiencies are common among Bangladeshi children, whose complementary diets frequently cannot affordably meet recommended micronutrient requirements [51]. Fourth, lead-associated immune dysfunction could contribute to a cycle of microbial infection, diarrhoea, and undernutrition [52]. This pathway may be reinforced in Bangladesh by concurrent arsenic exposure from groundwater, which has itself been associated with wasting and underweight in rural Bangladeshi children [53] and co-occurred with lead in the exposure patterns observed in this sample. Because of the cross-sectional design, the temporal direction and relative contribution of these pathways cannot be established here. Longitudinal studies with repeated measures of lead exposure, gut health, micronutrient status, and growth are needed to disentangle them in the Bangladeshi context.

This study has several strengths. It draws on the first nationally representative survey in Bangladesh to combine venous blood metal biomarkers with concurrent anthropometry, yielding a large analytic sample with survey-weighted estimates generalisable to the national child population. It also assessed lead’s association with growth after adjustment for co-exposure to arsenic, cadmium, and mercury and extended single-indicator analyses to composite anthropometric failure, identifying overlapping failure patterns associated with lead exposure. Several limitations also warrant consideration. First, the cross-sectional design means that exposure and outcomes were measured concurrently, so temporality cannot be established and the associations should not be interpreted as causal; reverse causation is possible, particularly for nutritional outcomes. Second, BLLs mainly reflect recent exposure and may not capture the cumulative exposure relevant to outcomes that develop over months to years, which could attenuate the associations if misclassification is non-differential. Third, residual confounding is possible because several established determinants were unavailable or largely missing in MICS7, including birth weight, dietary intake, gestational age and maternal anthropometry. Fourth, the analysis was restricted to complete cases (missing data by variable are given in Supplementary Table S15): 62.1% of the 17,132 blood-eligible children had a valid blood lead result, and those with a result differed from the others in age, residence, division, maternal education, household wealth and the prevalence of wasting, underweight and CIAF (Supplementary Table S16). Re-weighting the analytic sample by the inverse probability of inclusion left the main estimates essentially unchanged (Supplementary Tables S7 and S8), but this can account only for observed characteristics, so selection bias cannot be excluded. The 166 children excluded after a recorded blood result were too few to assess differences reliably and included more children aged 12–23 months (Supplementary Table S1). Fifth, estimates for arsenic, cadmium and mercury come from models built to test the robustness of the lead association and should not be interpreted as independent effects of those metals (Supplementary Table S3), and interactions between metals were not evaluated. In particular, the associations of arsenic with higher WAZ and WHZ run opposite to the expected direction and more probably reflect residual confounding than a protective effect. Sixth, seven outcomes were examined without correction for multiple testing, and the tests for non-linearity and a threshold have limited power to detect modest departures. Finally, laboratory accreditation and quality-control results were not available in the MICS7 documentation, so some measurement error cannot be excluded.

Bangladesh has already established several platforms relevant to lead exposure, including clinical guidance for lead poisoning, environmental regulations governing ULAB recycling, and national biomarker surveillance through MICS 2025 [5,24,25]. The observed inverse associations between BLLs and anthropometric indicators indicate that lead exposure should be considered within the country’s child growth and nutrition agenda. Rather than universal screening, which is constrained by the limited number of reference laboratories able to measure BLLs [24], blood lead testing and environmental investigation could be prioritised in the highest-burden districts and in communities with known or suspected sources, such as informal ULAB recycling and contaminated food or consumer products, pending confirmation with more granular district-level estimates. Linking this with growth monitoring at existing maternal and child health contact points and with environmental source control may identify high-risk children earlier and be more efficient than nutrition-focused interventions alone. The present determinants analysis offers additional, actionable targets for source-control efforts. Household soil lead testing, prioritised in areas near informal battery-recycling and industrial sites, could identify homes where soil remediation, already shown to reduce children’s BLLs at contaminated point sources [27,28], would most benefit exposed children. Occupational health measures for household members engaged in battery manufacturing, repair, or recycling, jewellery recycling, e-waste processing, or automobile repair, such as workplace hygiene practices, wearing protective clothing, and avoiding take-home contamination through changing clothes and washing before contact with children, could reduce household-level exposure from these informal industries. Given the association between pica (ingestion of dirt or paint chips) and elevated BLLs, caregiver education on safe play environments and handwashing, together with attention to iron deficiency, a known risk factor for pica, may help reduce dirt and paint-chip ingestion, particularly in homes with peeling or cracked paint or elevated soil lead. Finally, a coordinated approach linking source control, environmental surveillance, food-safety monitoring, clinical management, and population-level biomonitoring could strengthen Bangladesh’s existing systems for addressing childhood lead exposure and its potential contribution to poor growth.

## 5. Conclusions

In this nationally representative sample of Bangladeshi children, BLLs were independently associated with impaired linear and ponderal growth, elevated stunting, wasting, and underweight prevalence, and specific patterns of composite anthropometric failure, particularly those involving stunting. Household soil lead concentration, occupational contact with lead-related work, and child pica behaviour emerged as significant, modifiable correlates of elevated BLLs, highlighting concrete targets for exposure-reduction efforts. These findings position lead exposure as a nutritionally relevant public health concern in Bangladesh and support prioritising source-control interventions, integrating growth surveillance with environmental exposure monitoring.

## Figures and Tables

**Table 1:** Characteristics of the analytic sample of Bangladeshi children aged 12–59 months (*n* = 10,495).

Characteristic	Category	*n*	Weighted %
Elevated BLLs (≥5 μg/dL)	No	7163	61.4
	Yes	3332	38.6
Median BLLs (μg/dL) ^a^	-	10,495	4.3 (3.1, 6.2)
Median blood arsenic levels (μg/L) ^a^	-	10,495	1.9 (1.4, 2.9)
Median blood cadmium levels (μg/L) ^a^	-	10,495	0.4 (0.2, 0.6)
Median blood mercury levels (μg/L) ^a^	-	10,495	1.4 (0.9, 2.3)
Child sex	Male	5503	52.5
	Female	4992	47.5
Child age group	12–23	1982	18.8
	24–35	2684	26.0
	36–47	2916	27.0
	48–59	2913	28.2
Maternal education	Pre-primary/none	738	7.0
	Primary	2027	19.5
	Secondary	5716	54.6
	Higher	2014	18.8
Place of residence	Urban	2609	29.3
	Rural	7886	70.7
Division	Barishal	1079	5.2
	Chattogram	2102	23.2
	Dhaka	1765	24.1
	Khulna	1595	10.4
	Mymensingh	644	8.1
	Rajshahi	1135	11.4
	Rangpur	1505	11.3
	Sylhet	670	6.3
Household wealth index	Poorest	2793	22.2
	Poorer	2476	21.5
	Middle	2117	20.2
	Richer	1835	19.8
	Richest	1274	16.3

^a^ Values were reported as survey-weighted median (IQR).

**Table 2:** Distribution of stunting, wasting, underweight, and CIAF by background characteristics (*n* = 10,495).

Characteristic	Category	Stunting, *n* (%)	*p* Value	Wasting, *n* (%)	*p* Value	Underweight, *n* (%)	*p* Value	CIAF, *n* (%)	*p* Value
Overall	-	2717 (25.9)	-	1496 (14.1)	-	2800 (26.5)	-	4040 (38.2)	-
Elevated BLLs (≥5 µg/dL)	No	1772 (24.8)	0.014	1014 (14.1)	0.930	1877 (26.4)	0.780	2694 (37.4)	0.102
	Yes	945 (27.5)		482 (14.2)		923 (26.7)		1346 (39.4)	
Child sex	Male	1432 (25.9)	0.854	843 (15.2)	0.004	1462 (26.9)	0.428	2119 (38.5)	0.567
	Female	1285 (25.8)		653 (12.9)		1338 (26.1)		1921 (37.8)	
Child age group	12–23	553 (27.8)	<0.001	261 (13.4)	0.018	482 (23.9)	0.034	756 (37.9)	0.583
	24–35	763 (28.1)		344 (12.4)		714 (26.3)		1070 (39.5)	
	36–47	757 (26.0)		427 (14.7)		777 (26.6)		1116 (38.0)	
	48–59	644 (22.3)		464 (15.7)		827 (28.5)		1098 (37.3)	
Maternal education	Pre-primary/none	266 (35.8)	<0.001	110 (14.0)	<0.001	248 (32.8)	<0.001	338 (45.1)	<0.001
	Primary	671 (33.4)		324 (16.2)		658 (32.2)		923 (45.2)	
	Secondary	1419 (24.8)		851 (14.5)		1500 (26.0)		2187 (37.9)	
	Higher	361 (17.3)		211 (10.9)		394 (20.0)		592 (29.2)	
Place of residence	Urban	620 (22.9)	<0.001	360 (13.5)	0.343	644 (24.1)	0.006	940 (35.3)	0.003
	Rural	2097 (27.1)		1136 (14.4)		2156 (27.6)		3100 (39.4)	
Division	Barishal	280 (26.6)	<0.001	128 (12.0)	0.001	276 (25.9)	<0.001	410 (38.3)	<0.001
	Chattogram	579 (27.3)		308 (15.5)		596 (29.0)		844 (40.4)	
	Dhaka	419 (21.9)		212 (11.7)		378 (19.8)		612 (32.4)	
	Khulna	357 (22.9)		226 (14.2)		410 (26.4)		564 (36.0)	
	Mymensingh	181 (28.7)		84 (11.9)		198 (31.5)		266 (41.3)	
	Rajshahi	287 (25.2)		203 (17.3)		320 (27.9)		452 (39.0)	
	Rangpur	358 (24.2)		223 (14.9)		389 (26.3)		560 (37.6)	
	Sylhet	256 (40.0)		112 (15.8)		233 (35.4)		332 (50.9)	
Household wealth index	Poorest	930 (34.6)	<0.001	435 (15.3)	0.001	917 (33.7)	<0.001	1273 (46.3)	<0.001
	Poorer	679 (27.7)		393 (16.3)		743 (30.3)		1041 (42.0)	
	Middle	513 (25.2)		282 (13.3)		513 (25.6)		765 (36.6)	
	Richer	409 (23.4)		243 (14.0)		415 (24.1)		632 (36.3)	
	Richest	186 (15.3)		143 (10.8)		212 (16.0)		329 (26.2)	

*n* represents the unweighted number of children. Percentages are survey-weighted row percentages within each background-characteristic category. *p* values were from Rao–Scott chi-square tests.

**Table 3:** Associations of BLLs with continuous anthropometric outcomes.

Outcome	Model 1 Adjusted β (95% CI)	*p* Value	Model 2 Adjusted β (95% CI)	*p* Value
HAZ	−0.075 (−0.103, −0.046)	<0.001	−0.078 (−0.107, −0.048)	<0.001
WAZ	−0.062 (−0.088, −0.036)	<0.001	−0.064 (−0.091, −0.037)	<0.001
WHZ	−0.029 (−0.055, −0.003)	0.031	−0.029 (−0.056, −0.002)	0.034

Note: Values were adjusted β coefficients and 95% confidence intervals from linear regression models. β represents the adjusted mean difference in the outcome associated with a 1-SD increase in the natural log-transformed concentration of the specified metal. HAZ = height-for-age z-score; WAZ = weight-for-age z-score; WHZ = weight-for-height z-score; CI = confidence interval; SD = standard deviation. Model 1 included BLLs as exposure and was adjusted for covariates (child sex, child age, maternal education, place of residence, division, and household wealth). Model 2 is a mutually metal-adjusted model including BLLs as exposure and arsenic, cadmium, and mercury as co-occurring exposures and adjusting for covariates (child sex, child age, maternal education, place of residence, division, and household wealth).

**Table 4:** Associations of BLLs with binary anthropometric outcomes.

Outcome	Model 1: Adjusted PR (95% CI)	*p* Value	Model 2: Adjusted PR (95% CI)	*p* Value
Stunting	1.09 (1.05, 1.14)	<0.001	1.10 (1.05, 1.14)	<0.001
Wasting	1.08 (1.02, 1.15)	0.012	1.09 (1.02, 1.16)	0.011
Underweight	1.08 (1.04, 1.13)	<0.001	1.08 (1.04, 1.13)	<0.001
CIAF	1.07 (1.04, 1.11)	<0.001	1.08 (1.04, 1.11)	<0.001

Note: Values were adjusted prevalence ratios (PRs) and 95% confidence intervals from modified Poisson regression models. PRs represent the ratio of outcome prevalence associated with a 1-SD increase in the natural log-transformed concentration of the specified metal. PR = prevalence ratio; CI = confidence interval; SD = standard deviation. Model 1 included BLLs as exposure and was adjusted for covariates (child sex, child age, maternal education, place of residence, division, and household wealth). Model 2 is a mutually metal-adjusted model including BLLs as exposure and arsenic, cadmium, and mercury as co-occurring exposures and adjusting for covariates (child sex, child age, maternal education, place of residence, division, and household wealth).

**Table 5:** Associations of blood lead concentrations with specific patterns of composite anthropometric failure.

CIAF Category vs. No Failure ^a^	Model 1: Adjusted PR (95% CI)	*p* Value	Model 2: Adjusted PR (95% CI)	*p* Value
Wasting only	1.00 (0.86–1.17)	0.984	1.00 (0.85–1.18)	0.971
Wasting + underweight	1.14 (1.04–1.25)	0.008	1.14 (1.04–1.26)	0.007
Stunting + wasting + underweight	1.18 (1.06–1.31)	0.003	1.18 (1.06–1.32)	0.003
Stunting + underweight	1.11 (1.04–1.19)	0.002	1.11 (1.03–1.19)	0.003
Stunting only	1.12 (1.03–1.21)	0.007	1.13 (1.04–1.22)	0.004
Underweight only	1.05 (0.91–1.20)	0.515	1.03 (0.89–1.20)	0.656

Note: Values were adjusted prevalence ratios (PRs) and 95% confidence intervals from modified Poisson regression models. PRs represent the ratio of outcome prevalence associated with a 1-SD increase in the natural log-transformed concentration of the specified metal. PR = prevalence ratio; CI = confidence interval; SD = standard deviation; CIAF = composite index of anthropometric failure. ^a^ Separate model for each of the six CIAF categories. Model 1 included BLLs as exposure and was adjusted for covariates (child sex, child age, maternal education, place of residence, division, and household wealth). Model 2 is a mutually metal-adjusted model including BLLs as exposure and arsenic, cadmium, and mercury as co-occurring exposures and adjusting for covariates (child sex, child age, maternal education, place of residence, division, and household wealth).

## Data Availability

The data presented in this study were derived from the 2025 Bangladesh Multiple Indicator Cluster Survey (MICS), which is publicly available from UNICEF’s MICS Data Archive (https://mics.unicef.org, accessed on 6 September 2026) following registration and data request procedures.

## Supplementary Materials

The following supporting information can be downloaded at https://www.mdpi.com/article/10.3390/foods15213911/s1: Figure S1: Sample selection procedure; Figure S2. Spearman correlations among blood metal concentrations (unweighted analysis); Figure S3. Adjusted association between blood lead levels and anthropometric outcomes, estimated with a three-knot restricted cubic spline; Figure S4. Adjusted association between blood lead levels and anthropometric outcomes with additional adjustment for arsenic, cadmium and mercury, estimated with a three-knot restricted cubic spline; Table S1. Comparison of analysed children with children excluded after a recorded blood result, Bangladesh MICS7; Table S2. Metal co-occurrence according to the number of metals in the highest exposure category; Table S3. Single-pollutant and mutually adjusted associations of arsenic, cadmium and mercury with anthropometric outcomes in Bangladeshi children aged 12–59 months; Table S4. Shape of the association between blood lead levels and anthropometric outcomes: restricted cubic spline analysis and tests for a step or slope change at 5 µg/dL in Bangladeshi children aged 12–59 months (*n* = 10,495); Table S5. Definitions and categorisation of variables included in this study; Table S6. District-level sample size, mean blood lead levels (µg/dL), and prevalence of elevated blood lead levels, stunting, wasting, underweight, and CIAF among Bangladeshi children aged 12–59 months (*n* = 10,495); Table S7. Comparison of the analytic sample (*n* = 10,495) with all blood-eligible children under the original blood weight and inverse-probability weights, Bangladesh MICS7; Table S8. Sensitivity analysis: associations of blood lead levels with anthropometric outcomes under the original blood weight and inverse-probability weights in Bangladeshi children aged 12–59 months, *n* = 10,495; Table S9. Household, environmental, and demographic determinants of elevated BLLs (≥5 µg/dL) among children aged 12–59 months, Bangladesh; Table S10. Associations of elevated BLLs (≥5 µg/dL) with anthropometric outcomes; Table S11. Associations of elevated BLLs (≥5 µg/dL) with binary anthropometric outcomes; Table S12. Associations of elevated BLLs (≥5 µg/dL) with specific patterns of composite anthropometric failure; Table S13. Sensitivity analysis of the association between solid-fuel cooking and elevated blood lead levels (BLLs ≥ 5 µg/dL): stepwise adjustment for socioeconomic and geographic factors in Bangladeshi children aged 12–59 months (*n* = 10,277); Table S14. Weighted prevalence of solid-fuel cooking and of elevated BLLs by division in Bangladeshi children aged 12–59 months (children with non-missing cooking-fuel data, *n* = 10,494); Table S15. Missing data for the analysis variables among children aged 12–59 months, Bangladesh MICS7; Table S16. Sociodemographic and anthropometric comparison of age-eligible children (12–59 months) with and without a valid blood lead result, Bangladesh MICS7.
