# Supplementary material for: Lead Exposure and Nutritional Status Among Bangladeshi Children: An Analysis Based on Bangladesh Multiple Indicator Cluster Survey
Source: Foods. 2026 Oct 31;15(21):3911. doi: 10.3390/foods15213911 (PMC13762794; doi:10.3390/foods15213911)
Supplement: Supplementary file 1 [file foods-15-03911-s001.zip › foods-4622087-supplementary.pdf]

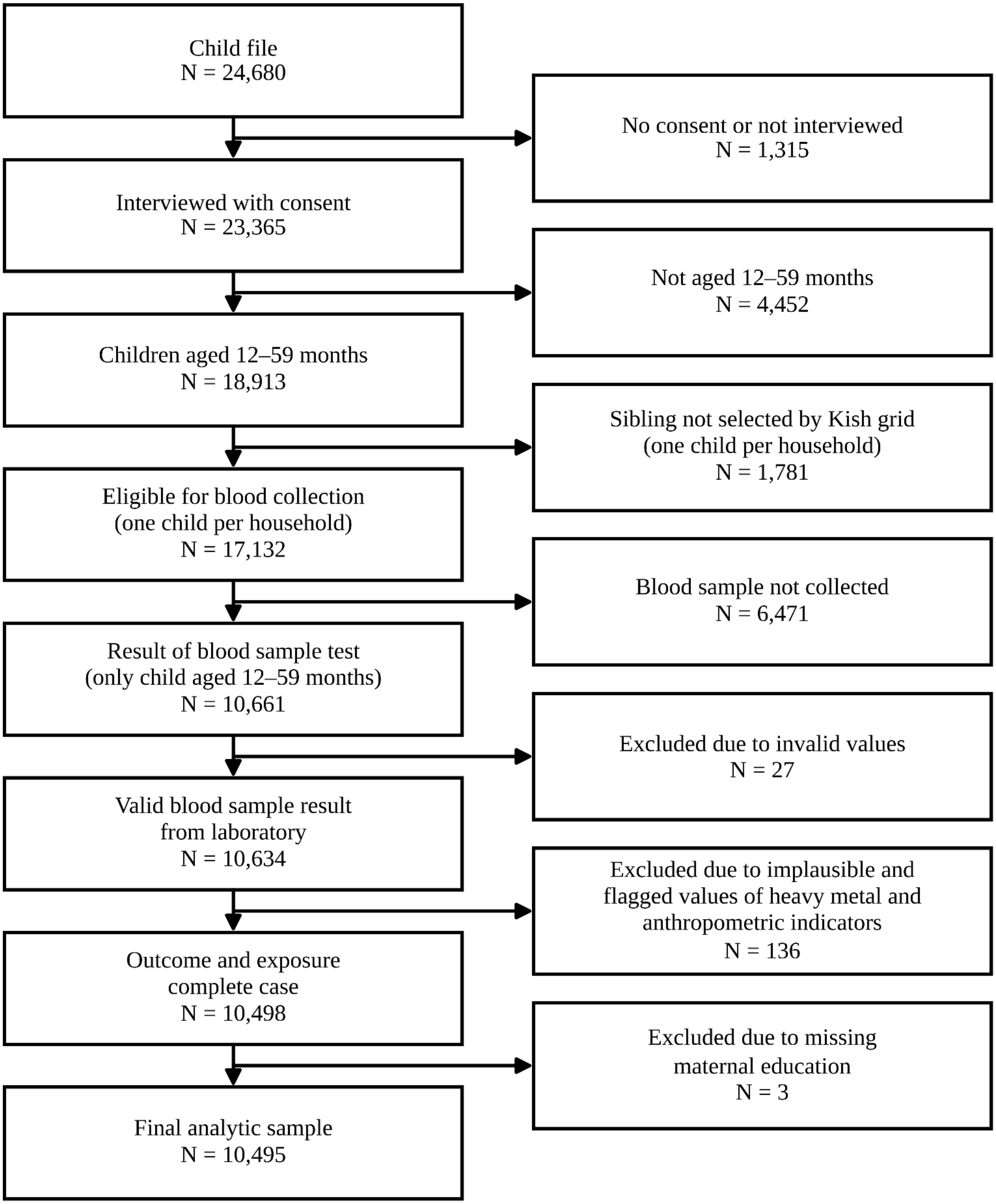

Figure S1. Sample selection procedure.

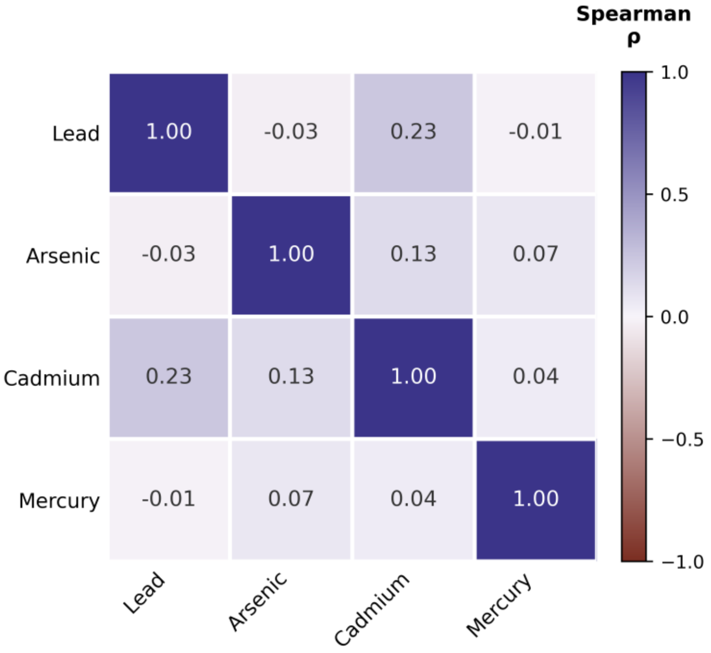

Figure S2. Spearman correlations among blood metal concentrations (unweighted analysis).

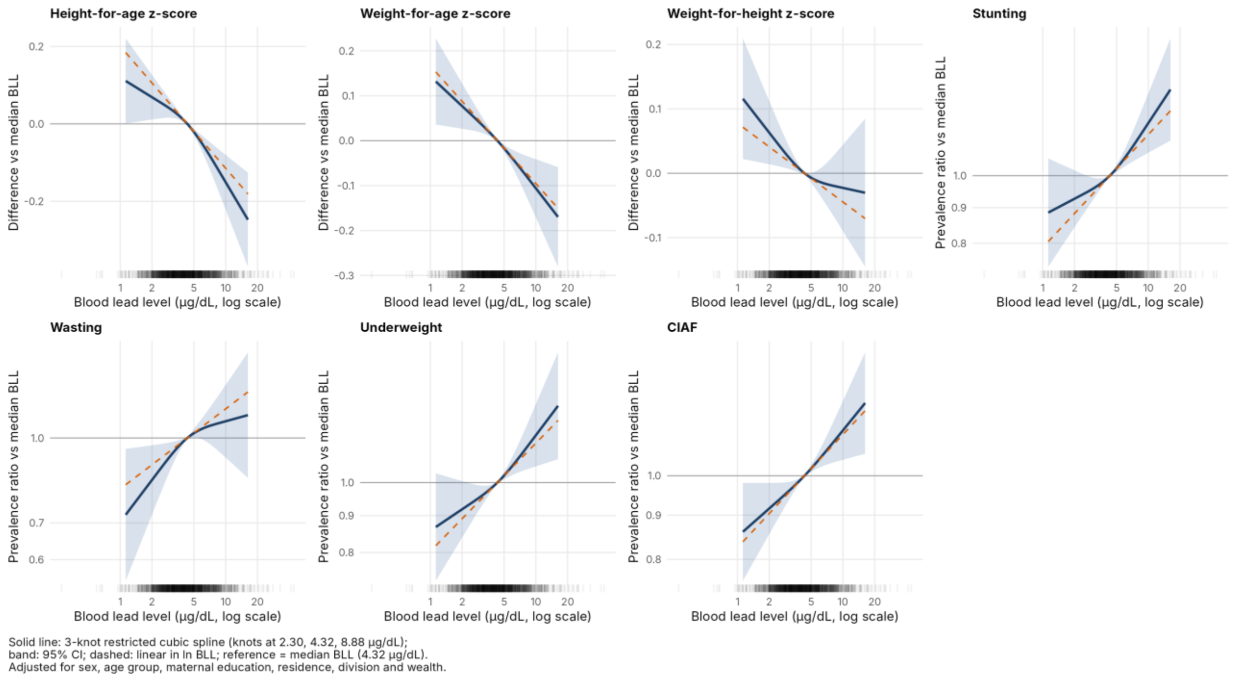

Figure S3. Adjusted association between blood lead levels and anthropometric outcomes, estimated with a three-knot restricted cubic spline. *The solid line and shaded band show the difference in z-score or the prevalence ratio (with 95% confidence interval) relative to a child at the median BLLs (4.32 µg/dL, the survey-weighted median); the dashed line shows the linear fit in ln BLLs, and the rug marks observed values. Model 1 of Table S4 (lead only).*

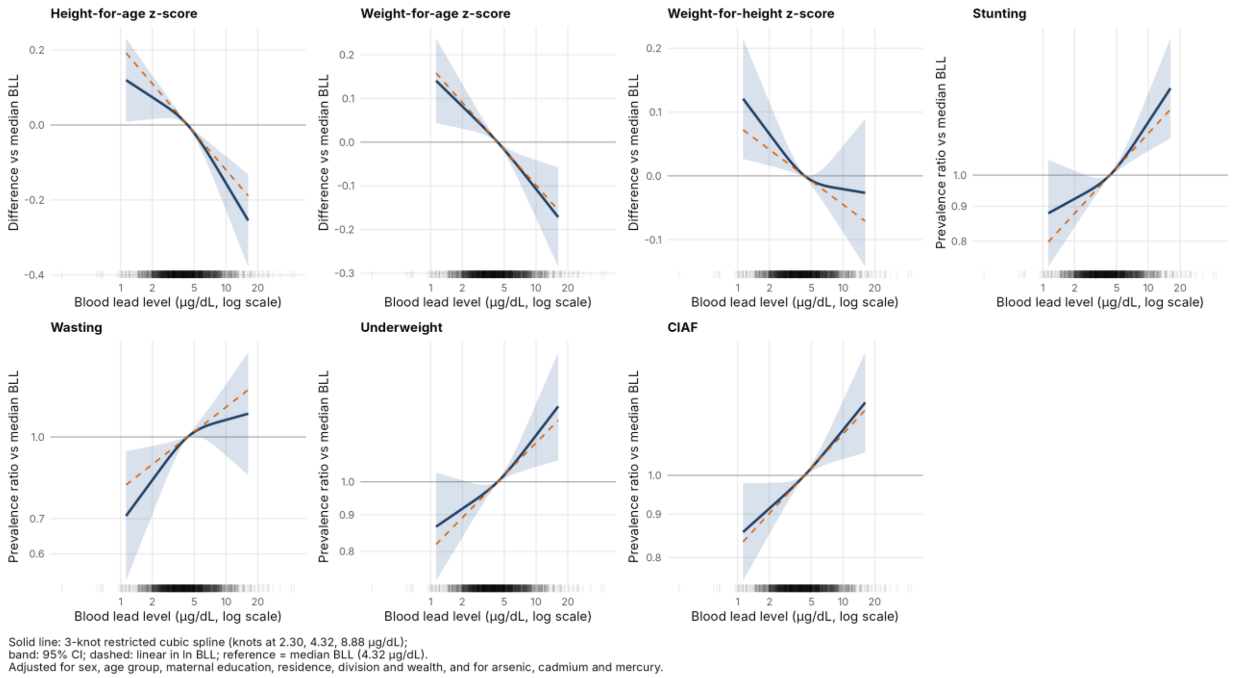

Figure S4. Adjusted association between blood lead levels and anthropometric outcomes with additional adjustment for arsenic, cadmium and mercury, estimated with a three-knot restricted cubic spline. *Elements are as in Figure S3: the solid line and shaded band show the difference in z-score or the prevalence ratio (with 95% confidence interval) relative to a child at the median BLLs (4.32 µg/dL, the survey-weighted median), the dashed line shows the linear fit in ln BLLs, and the rug marks observed values. Model 2 of Table S4 (lead, arsenic, cadmium and mercury).*

Table S1. Comparison of analysed children with children excluded after a recorded blood result, Bangladesh MICS7.

Characteristic	Category	Analysed (n=10,495), n (%)	Excluded after a blood result (n=166), n (%)	P value
Elevated BLLs (≥5 µg/dL)	Yes	3,332 (38.6)	35 (29.1)	0.085
Child sex	Male	5,503 (52.5)	86 (52.6)	0.987
	Female	4,992 (47.5)	80 (47.4)	
Child age group (months)	12-23	1,982 (18.8)	51 (32.3)	0.001
	24-35	2,684 (26.0)	51 (27.8)	
	36-47	2,916 (27.0)	26 (21.6)	
	48-59	2,913 (28.2)	38 (18.3)	
Maternal education	Pre-primary/none	738 (7.0)	17 (11.0)	0.206
	Primary	2,027 (19.5)	26 (15.1)	
	Secondary	5,716 (54.6)	86 (49.4)	
	Higher	2,014 (18.8)	34 (20.6)	
Place of residence	Urban	2,609 (29.3)	45 (32.6)	0.489
	Rural	7,886 (70.7)	121 (67.4)	
Division	Barishal	1,079 (5.2)	10 (2.3)	0.096
	Chattogram	2,102 (23.2)	29 (17.4)	
	Dhaka	1,765 (24.1)	34 (30.4)	
	Khulna	1,595 (10.4)	24 (9.5)	
	Mymensingh	644 (8.1)	15 (13.7)	
	Rajshahi	1,135 (11.4)	18 (9.4)	
	Rangpur	1,505 (11.3)	24 (9.8)	
	Sylhet	670 (6.3)	12 (7.6)	
Household wealth index	Poorest	2,793 (22.2)	38 (20.2)	0.943
	Poorer	2,476 (21.5)	40 (22.9)	
	Middle	2,117 (20.2)	34 (18.7)	
	Richer	1,835 (19.8)	30 (19.7)	
	Richest	1,274 (16.3)	24 (18.4)	

n is the unweighted number of children; percentages are survey-weighted column percentages using the blood-specific weight. P values are from Rao–Scott chi-square tests. Excluded children: 27 with an invalid laboratory result, 136 with implausible, flagged or missing values and 3 with missing maternal education (Figure S1). Elevated BLLs are based on the 127 excluded children with a valid lead value; the 3 children with missing maternal education are not shown for that variable.

Table S2. Metal co-occurrence according to the number of metals in the highest exposure category.

Number of metals in the highest exposure category	Percentage (%)
0 (none)	27.8
1	39.8
2	24.2
3	7.2
4 (all metals)	0.9

The highest exposure category was defined as blood lead ≥5 µg/dL (elevated BLLs) for lead and as the highest survey-weighted quartile of the distribution for arsenic, cadmium and mercury. Percentages are survey-weighted (blood-specific weight). If the highest quartile were used for lead as well, the distribution would be 33.6%, 39.5%, 21.0%, 5.3% and 0.6% for 0, 1, 2, 3 and 4 metals, respectively.

Table S3. Single-pollutant and mutually adjusted associations of arsenic, cadmium and mercury with anthropometric outcomes, Bangladeshi children aged 12–59 months.

Metal	Outcome	Single-pollutant β (95% CI)[a]	P value	Mutually-adjusted β (95% CI)[b]	P value
Arsenic	HAZ	0.013 (-0.014, 0.040)	0.346	0.008 (-0.020, 0.035)	0.585
Arsenic	WAZ	0.041 (0.016, 0.066)	0.001	0.035 (0.010, 0.060)	0.006
Arsenic	WHZ	0.050 (0.025, 0.076)	<0.001	0.046 (0.021, 0.072)	<0.001
Cadmium	HAZ	0.008 (-0.020, 0.037)	0.571	0.021 (-0.008, 0.051)	0.159

Cadmium	WAZ	0.017 (-0.010, 0.043)	0.220	0.024 (-0.003, 0.052)	0.081
Cadmium	WHZ	0.017 (-0.009, 0.044)	0.199	0.018 (-0.009, 0.045)	0.200
Mercury	HAZ	0.009 (-0.018, 0.035)	0.535	0.005 (-0.022, 0.032)	0.711
Mercury	WAZ	0.020 (-0.006, 0.046)	0.132	0.015 (-0.011, 0.041)	0.255
Mercury	WHZ	0.021 (-0.005, 0.047)	0.107	0.017 (-0.009, 0.043)	0.200
**Metal**	**Outcome**	**Single-pollutant PR (95% CI)[a]**	**P value**	**Mutually-adjusted PR (95% CI)[b]**	**P value**
Arsenic	Stunting	1.00 (0.96, 1.04)	0.950	1.00 (0.96, 1.05)	0.907
Arsenic	Wasting	0.94 (0.88, 1.00)	0.063	0.95 (0.89, 1.01)	0.110
Arsenic	Underweight	0.96 (0.92, 0.99)	0.027	0.96 (0.92, 1.00)	0.045
Arsenic	CIAF	0.98 (0.95, 1.01)	0.245	0.99 (0.96, 1.02)	0.347
Cadmium	Stunting	1.00 (0.96, 1.04)	1.000	0.98 (0.94, 1.03)	0.501
Cadmium	Wasting	0.96 (0.91, 1.03)	0.254	0.96 (0.89, 1.02)	0.174
Cadmium	Underweight	1.00 (0.96, 1.05)	0.916	0.99 (0.95, 1.04)	0.717
Cadmium	CIAF	1.00 (0.97, 1.03)	0.985	0.99 (0.96, 1.02)	0.502
Mercury	Stunting	1.01 (0.97, 1.06)	0.509	1.02 (0.98, 1.06)	0.418
Mercury	Wasting	0.97 (0.92, 1.03)	0.399	0.98 (0.92, 1.04)	0.525
Mercury	Underweight	0.99 (0.95, 1.03)	0.651	1.00 (0.96, 1.04)	0.852
Mercury	CIAF	1.00 (0.97, 1.04)	0.775	1.01 (0.98, 1.04)	0.609

Values are adjusted β coefficients (HAZ, WAZ, WHZ; linear regression) or adjusted prevalence ratios (PRs; stunting, wasting, underweight, CIAF; modified Poisson regression) with 95% confidence intervals per 1-SD increase in the natural log-transformed metal concentration. β represents the adjusted mean difference in z-score. All models accounted for the survey design (PSU, stratum, blood-specific weight). HAZ = height-for-age z-score; WAZ = weight-for-age z-score; WHZ = weight-for-height z-score; PR = prevalence ratio; CI = confidence interval; SD = standard deviation; CIAF = composite index of anthropometric failure.

[a] *Single-pollutant models included one metal as exposure and were adjusted for covariates (child sex, child age, maternal education, place of residence, division, and household wealth).*

[b] *Mutually adjusted models included lead, arsenic, cadmium, and mercury (each as natural log-transformed concentration standardized to 1 SD) as co-occurring exposures, and were adjusted for the same covariates.*

Table S4. Shape of the association between blood lead levels and anthropometric outcomes: restricted cubic spline analysis and tests for a step or slope change at 5 µg/dL, Bangladeshi children aged 12–59 months (N=10,495).

	Model 1				Model 2			
**Outcome**	**P, any association**	**P, non-linearity**	**P, step at 5 µg/dL**	**P, slope change at 5 µg/dL**	**P, any association**	**P, non-linearity**	**P, step at 5 µg/dL**	**P, slope change at 5 µg/dL**
Height-for-age z-score	<0.001	0.141	0.826	0.240	<0.001	0.147	0.827	0.253
Weight-for-age z-score	<0.001	0.630	0.300	0.761	<0.001	0.691	0.264	0.838
Weight-for-height z-score	0.025	0.323	0.234	0.345	0.023	0.273	0.191	0.288
Stunting	<0.001	0.251	0.875	0.288	<0.001	0.245	0.892	0.280
Wasting	0.025	0.315	0.357	0.259	0.021	0.283	0.340	0.226
Underweight	<0.001	0.448	0.554	0.527	0.001	0.470	0.521	0.554
CIAF	<0.001	0.669	0.721	0.652	<0.001	0.673	0.714	0.656

ln BLLs were modelled as a restricted cubic spline with three knots at the survey-weighted (blood-specific weight) 10th, 50th and 90th percentiles of its distribution (ln BLL 0.83, 1.46 and 2.18, corresponding to 2.30, 4.32 and 8.88 µg/dL). Model 1 included BLLs as exposure and were was adjusted for child sex, child age group, maternal education, place of residence, division and household wealth; Model 2 was additionally adjusted for arsenic, cadmium and mercury (standardised ln concentrations). Both accounted for the survey design (PSU, stratum and blood-specific weight); z-scores were analysed by linear regression and binary outcomes by modified Poisson regression. P values are design-based Wald tests. Any association: linear and non-linear spline terms tested together. Non-linearity: non-linear spline term alone. Step at 5 µg/dL: coefficient of an indicator for BLLs ≥5 µg/dL added to ln BLLs, i.e. a discontinuity in the outcome at 5 µg/dL beyond the linear trend (Model 1: WHZ difference 0.05 (95% CI -0.03 to 0.13); wasting prevalence ratio 0.91 (95% CI 0.76 to 1.11)). Slope change at 5 µg/dL: coefficient of max(ln BLL − ln 5, 0) added to ln BLLs, i.e. a change in the slope above 5 µg/dL (Model 1: WHZ 0.07 (95% CI -0.07 to 0.21) per unit ln BLLs; wasting prevalence ratio 0.82 (95% CI 0.58 to 1.16)). A non-significant P value indicates no detectable departure or break, not proof of its absence, and the power to detect small departures is limited. The fitted curves are

shown in Figure S3 (Model 1) and Figure S4 (Model 2). BLL = blood lead level; CIAF = composite index of anthropometric failure.

Table S5. Definitions and categorisation of variables included in this study.

Variable	Definition / construction	Categories
Household soil lead concentration	Directly sampled household soil lead concentration (mg/kg). Values coded 0 or 999 (non-detect/missing sentinel codes) set to missing. Right-skewed; categorised into survey-weighted quartiles (blood-specific weight) (Q1 lowest – Q4 highest). Also modelled continuously as the natural log, standardised to a z-score (per 1-SD increment).	Quartile (Q1–Q4); continuous z-score of ln(soil lead) in sensitivity analysis
Neighbourhood informal lead-related industry proximity	Composite of six household-reported yes/no items on nearby informal industrial activity (e.g., battery/metal recycling, smelting, welding). Summed to a count of activities reported (0–6), then collapsed to reduce sparse cells.	0 activities; 1 activity; ≥2 activities
Household occupational contact with lead-related work	Whether any household member with regular child contact works in battery manufacturing, repair, or recycling, jewellery recycling, e-waste recycling, or automobile repair. Coded Yes if any of the six items = Yes; No if all non-missing items = No.	No; Yes
Condition of paint on household walls/windows/doors	Household-reported condition of interior/exterior paint.	No paint; Good condition; Peeling/chipping/cracking
Child's pica behaviour	Caregiver-reported frequency of the child ingesting non-food items — dirt or pieces of paint — in the last 3 months. Value 9 (don't know/missing) set to missing.	Not at all; Less than weekly; At least weekly; Almost daily
Solid fuel used for cooking	Household's primary cooking fuel, classified as solid (wood, straw, dung, crop residue, charcoal, coal) versus clean/non-solid.	No; Yes
Improved sanitation facility	Household sanitation facility type classified per the WHO/UNICEF Joint Monitoring Programme improved/unimproved definitions.	Unimproved; Improved
Child sex	Child's sex as recorded in the household roster.	Male; Female

Child age group	Child's age in completed months at interview, grouped into four bands.	12–23 months; 24–35 months; 36–47 months; 48–59 months
Maternal education	Highest education level completed by the child's mother or primary caregiver.	Pre-primary/none; Primary; Secondary; Higher
Residence	Household's urban/rural classification per the survey's sampling frame.	Urban; Rural
Division	Administrative division of household residence.	Barishal; Chattogram; Dhaka; Khulna; Mymensingh; Rajshahi; Rangpur; Sylhet
Household wealth quintile	Household wealth index quintile, constructed from asset ownership and housing characteristics via principal components analysis (standard MICS wealth index construction).	Poorest; Poorer; Middle; Richer; Richest

Elevated BLLs (outcome) was defined as BLLs ≥5 μg/dL, consistent with the MICS7 protocol threshold and the definition used throughout the main manuscript.

Table S6. District-level sample size, mean blood lead levels (µg/dL), and prevalence of elevated blood lead levels, stunting, wasting, underweight, and CIAF among Bangladeshi children aged 12–59 months (N=10,495).

District	n	Mean BLLs (µg/dL)	Elevated BLLs prevalence (≥5 µg/dL) (%)	Stunting prevalence (%)	Wasting prevalence (%)	Underweight prevalence (%)	CIAF prevalence (%)
Bagerhat	131	4.01	21.75	26.14	17.37	35.55	45.47
Bandarban	271	4.51	29.69	25.60	16.69	33.13	39.98
Barguna	188	4.52	33.45	25.90	11.82	23.81	38.88
Barisal	175	5.37	39.60	22.27	12.25	25.88	34.25
Bhola	202	4.39	26.73	34.49	12.93	26.56	42.65
Bogra	134	4.96	27.48	24.27	15.55	23.56	35.52
Brahamanbaria	165	6.16	50.62	31.47	16.71	29.60	41.79
Chandpur	176	5.86	54.40	23.43	13.84	26.36	34.66
Chittagong	277	6.12	48.20	28.72	21.60	34.65	45.12
Chuadanga	177	3.14	7.15	22.00	17.74	28.01	37.96
Comilla	141	5.07	44.74	21.89	14.86	25.62	36.42
Cox's Bazar	209	5.18	30.60	36.62	11.80	30.74	45.90
Dhaka	298	9.92	90.65	20.35	9.96	16.54	28.77
Dinajpur	153	3.73	15.93	22.82	15.81	25.39	37.96
Faridpur	126	5.47	37.92	29.05	20.37	33.28	44.16
Feni	201	4.38	28.56	31.74	13.28	27.40	43.15
Gaibandha	169	3.79	20.75	23.06	15.14	22.65	34.73
Gazipur	148	6.72	65.07	19.19	10.82	16.35	29.69
Gopalganj	120	4.59	26.30	31.16	11.43	24.45	41.16
Habiganj	226	5.59	48.31	30.07	16.80	28.72	43.97
Jamalpur	178	3.80	18.12	23.94	10.94	28.07	37.39
Jessore	155	3.39	12.15	25.59	11.17	29.88	39.52
Jhalokati	168	4.17	18.58	24.30	10.93	24.87	34.95
Jhenaidah	176	3.72	20.71	19.25	12.04	20.26	30.09
Joypurhat	112	4.32	27.84	20.91	22.25	25.77	40.21
Khagrachhari	127	5.47	40.17	23.36	11.50	21.10	31.69
Khulna	129	4.94	38.80	23.50	18.96	26.78	36.14
Kishoreganj	122	5.66	52.82	22.05	11.70	27.87	37.35

Kurigram	195	4.40	24.74	24.17	13.25	24.98	37.09
Kushtia	146	3.38	5.97	19.61	13.33	21.62	31.03
Lakshmipur	152	4.77	33.80	15.69	10.84	21.78	28.85
Lalmonirhat	212	3.63	18.08	22.77	13.41	27.30	38.02
Madaripur	136	4.91	42.15	22.22	12.01	16.28	34.07
Magura	208	3.24	10.89	28.49	11.71	26.13	37.61
Manikganj	96	6.28	47.60	17.33	3.72	11.29	22.22
Maulvibazar	144	5.79	47.05	31.70	15.95	31.65	42.50
Meherpur	132	2.57	6.08	20.77	12.34	19.62	32.53
Munshiganj	104	6.73	66.22	31.75	13.31	28.65	43.27
Mymensingh	156	5.27	40.01	30.03	10.86	33.71	42.29
Naogaon	109	3.71	14.64	33.63	15.70	31.89	44.69
Narail	196	3.97	20.08	21.00	14.26	27.70	34.09
Narayanganj	119	8.79	93.73	21.03	12.50	13.28	29.55
Narsingdi	113	6.79	64.79	18.78	10.36	15.40	27.39
Natore	140	3.28	4.43	25.07	22.18	29.39	42.28
Nawabganj	196	3.16	8.11	24.86	13.46	24.60	36.24
Netrakona	156	4.33	30.79	36.33	13.30	32.30	46.22
Nilphamari	213	4.08	25.10	21.36	17.59	28.66	37.35
Noakhali	269	4.72	36.50	30.45	11.68	29.42	42.45
Pabna	156	3.43	16.14	28.77	15.35	29.90	39.80
Panchagarh	199	3.32	10.51	26.40	12.74	23.50	36.00
Patuakhali	173	4.08	21.56	28.23	12.79	30.08	44.63
Pirojpur	173	4.14	23.28	20.52	9.83	22.34	31.71
Rajbari	159	4.01	22.65	24.72	7.84	20.05	34.34
Rajshahi	176	3.31	7.74	23.44	21.78	32.72	40.43
Rangamati	114	5.41	36.59	26.34	18.62	25.68	42.65
Rangpur	178	3.95	21.54	29.38	14.03	31.61	40.67
Satkhira	145	4.06	24.00	22.16	13.22	25.90	35.26
Shariatpur	132	5.03	46.77	27.79	17.43	31.64	43.95
Sherpur	154	3.88	19.02	22.04	16.35	28.23	38.07
Sirajganj	112	3.79	18.39	21.29	16.64	26.21	37.15
Sunamganj	196	5.39	50.66	42.73	15.02	42.09	52.98
Sylhet	104	5.44	44.52	49.44	15.75	36.89	58.69
Tangail	92	5.66	47.94	19.90	14.60	22.96	32.16

Thakurgaon	186	3.75	15.13	22.70	16.08	22.80	36.82

n is the unweighted number of children in each of the 64 districts; each city corporation was combined with its parent district (Dhaka North and Dhaka South with Dhaka; Chattogram City Corporation with Chattogram), as in Figure 1. Values are survey-weighted estimates accounting for PSU and stratum; all estimates used the blood-specific weight. The number of children per district ranged from 92 to 298 (median 156); two districts had fewer than 100 children (Manikganj, N=96; Tangail, N=92), and their estimates should be interpreted with caution. BLLs = blood lead levels; CIAF = composite index of anthropometric failure. Elevated BLLs was defined as ≥5 µg/dL; stunting, wasting and underweight as z-scores below −2 SD.

Table S7. Comparison of the analytic sample (N=10,495) with all blood-eligible children under the original blood weight and inverse-probability weights, Bangladesh MICS7.

Characteristic	Category	All eligible children (target)	Analytic sample, original blood weight	Analytic sample, IPW-A	Analytic sample, IPW-B
Child sex	Male	52.0	52.5	51.9	51.8
	Female	48.0	47.5	48.1	48.2
Child age group (months)	12-23	24.0	18.8	23.5	23.3
	24-35	26.1	26.0	26.3	26.3
	36-47	25.6	27.0	25.8	25.9
	48-59	24.3	28.2	24.4	24.5
Maternal education	Pre-primary/none	6.5	7.0	6.5	6.5
	Primary	18.2	19.5	18.2	18.1
	Secondary	53.4	54.6	53.4	53.9
	Higher	21.9	18.8	21.8	21.5
Place of residence	Urban	29.2	29.3	29.1	29.1
	Rural	70.8	70.7	70.9	70.9
Division	Barishal	5.3	5.2	5.3	5.4
	Chattogram	22.9	23.2	22.8	22.8

	Dhaka	24.2	24.1	24.1	24.2
	Khulna	10.7	10.4	10.7	10.8
	Mymensingh	8.4	8.1	8.3	8.0
	Rajshahi	11.6	11.4	11.7	11.8
	Rangpur	11.0	11.3	11.0	11.0
	Sylhet	6.0	6.3	6.0	5.9
Household wealth index	Poorest	20.8	22.2	20.8	20.7
	Poorer	20.0	21.5	20.2	20.3
	Middle	19.8	20.2	19.8	19.7
	Richer	20.4	19.8	20.4	20.4
	Richest	19.0	16.3	18.8	18.9

Values are survey-weighted percentages. Target population: the 17,120 children aged 12–59 months eligible for blood collection with known age, maternal education and household wealth (general child weight); 12 eligible children with one of these characteristics unknown, none of whom was analysed, are not included. Analytic sample: 10,495 children with a valid blood lead result and complete outcome and covariate data, weighted by the original blood-specific weight or by inverse-probability weights (IPW). IPW was calculated as the general child weight divided by the estimated probability that an eligible child was included in the analytic sample, truncated at the 1st and 99th percentiles (truncation limits 0.158–5.871 for IPW-A and 0.155–5.644 for IPW-B); inclusion probabilities were estimated by survey-weighted logistic regression and treated as fixed. IPW-A (primary sensitivity analysis) used only characteristics observed before selection: sampling stratum (each stratum lies within a single residence and division, so both are captured), child age group, sex, maternal education, household wealth quintile and the number of age-eligible children in the household. IPW-B (exploratory) additionally included the WHO height-for-age, weight-for-age and weight-for-height z-scores and was fitted among the 16,288 children with all three valid z-scores (including all 10,495 analysed children); because it conditions on anthropometry, it should not be interpreted as a conventional outcome-independent correction.

Table S8. Sensitivity analysis: associations of blood lead levels with anthropometric outcomes under the original blood weight and inverse-probability weights, Bangladeshi children aged 12–59 months, N=10,495.

	**Per 1-SD increase in ln BLLs**			**Elevated BLLs (≥5 µg/dL) vs. <5 µg/dL**		
**Outcome**	**Original**	**IPW-A**	**IPW-B**	**Original**	**IPW-A**	**IPW-B**
HAZ, β (95% CI)	−0.075 (−0.103, −0.046)	−0.073 (−0.102, −0.044)	−0.073 (−0.102, −0.044)	−0.105 (−0.166, −0.044)	−0.111 (−0.173, −0.048)	−0.112 (−0.174, −0.050)

WAZ, β (95% CI)	−0.062 (−0.088, −0.036)	−0.064 (−0.090, −0.037)	−0.065 (−0.091, −0.038)	−0.071 (−0.127, −0.014)	−0.080 (−0.138, −0.022)	−0.085 (−0.144, −0.026)
WHZ, β (95% CI)	−0.029 (−0.055, −0.003)	−0.033 (−0.060, −0.007)	−0.035 (−0.061, −0.008)	−0.018 (−0.077, 0.040)	−0.029 (−0.089, 0.031)	−0.035 (−0.096, 0.026)
Stunting, APR (95% CI)	1.09 (1.05, 1.14)	1.09 (1.04, 1.14)	1.09 (1.04, 1.13)	1.13 (1.04, 1.23)	1.14 (1.05, 1.24)	1.14 (1.04, 1.23)
Wasting, APR (95% CI)	1.08 (1.02, 1.15)	1.09 (1.02, 1.16)	1.09 (1.02, 1.16)	1.08 (0.94, 1.23)	1.09 (0.96, 1.25)	1.10 (0.97, 1.25)
Underweight, APR (95% CI)	1.08 (1.04, 1.13)	1.08 (1.04, 1.13)	1.09 (1.04, 1.13)	1.11 (1.01, 1.21)	1.11 (1.01, 1.21)	1.11 (1.02, 1.21)
CIAF, APR (95% CI)	1.07 (1.04, 1.11)	1.07 (1.04, 1.11)	1.07 (1.04, 1.11)	1.10 (1.03, 1.17)	1.11 (1.04, 1.18)	1.11 (1.04, 1.19)
Elevated BLL prevalence, % (95% CI)	38.6 (37.3–40.0)	38.5 (37.2–39.8)	38.4 (37.1–39.7)			

Values are adjusted β coefficients (HAZ, WAZ, WHZ; linear regression) or adjusted prevalence ratios (APRs; stunting, wasting, underweight, CIAF; modified Poisson regression) with 95% confidence intervals. β represents the adjusted mean difference in z-score. Exposures were ln BLLs (per 1-SD increase) or elevated BLLs (≥5 µg/dL vs. <5 µg/dL); the last row is the weighted prevalence of elevated BLLs. All models were adjusted for child sex, child age, maternal education, place of residence, division, and household wealth, and accounted for the survey design (PSU, stratum; t-based 95% confidence intervals). Original = blood-specific weight; IPW-A and IPW-B are defined in Table S7. Kish effective sample size: 7,012 (original), 6,904 (IPW-A), 6,934 (IPW-B). HAZ = height-for-age z-score; WAZ = weight-for-age z-score; WHZ = weight-for-height z-score; CIAF = composite index of anthropometric failure; BLLs = blood lead levels; CI = confidence interval; SD = standard deviation.

Table S9. Household, environmental, and demographic determinants of elevated BLLs (≥5 µg/dL) among children aged 12–59 months, Bangladesh.

Variables	n	Elevated BLLs, %†	Crude PR (95% CI)	P value	Adjusted PR (95% CI)‡	P value
Household soil lead concentration (quartile, mg/kg)						
Q1 (lowest)	2791	34.6	1.00 (ref)		1.00 (ref)	
Q2	2703	33.0	0.95 (0.86–1.06)	0.366	1.00 (0.91–1.10)	0.977
Q3	3015	33.8	0.98 (0.89–1.08)	0.677	1.03 (0.94–1.13)	0.527
Q4 (highest)	1902	54.4	1.57 (1.44–1.72)	<0.001	1.22 (1.11–1.33)	<0.001
Missing	84	75.8	–		–	
Neighbourhood informal lead-related industry activity nearby						
0	5164	35.2	1.00 (ref)		1.00 (ref)	
1	1539	35.0	0.99 (0.88–1.11)	0.904	1.01 (0.92–1.12)	0.807
2+	3697	44.1	1.25 (1.16–1.35)	<0.001	1.04 (0.96–1.12)	0.332
Missing	95	50.8	–		–	
Household member with child contact works in lead-related occupation (battery mfg/repair/recycling, e-waste or jewelry recycling, auto repair)						
No	9270	37.6	1.00 (ref)		1.00 (ref)	
Yes	1163	45.9	1.22 (1.11–1.34)	<0.001	1.08 (1.00–1.17)	0.049
Missing	62	44.1	–		–	
Condition of paint on household walls/windows/doors						
No paint	6208	33.0	1.00 (ref)		1.00 (ref)	
Good condition	3061	46.1	1.40 (1.31–1.50)	<0.001	1.02 (0.94–1.10)	0.633
Peeling/chipping/cracking	1226	42.9	1.30 (1.18–1.43)	<0.001	1.03 (0.94–1.13)	0.486
Child's pica behaviour (ingesting dirt or paint chips), frequency in last 3 months						
Not at all	9493	37.7	1.00 (ref)		1.00 (ref)	
Less than weekly	492	42.0	1.12 (0.97–1.28)	0.126	1.04 (0.91–1.20)	0.549

At least weekly	259	43.1	1.14 (0.96–1.36)	0.127	1.00 (0.86–1.16)	0.971
Almost daily	249	55.7	1.48 (1.29–1.69)	<0.001	1.18 (1.05–1.33)	0.006
Missing	2	100.0	–		–	
Solid fuel used for cooking						
No	1652	62.3	1.00 (ref)		1.00 (ref)	
Yes	8842	31.7	0.51 (0.48–0.54)	<0.001	0.78 (0.72–0.85)	<0.001
Missing	1	100.0	–		–	
Improved sanitation facility						
Unimproved	1138	46.5	1.00 (ref)		1.00 (ref)	
Improved	9357	37.6	0.81 (0.74–0.89)	<0.001	0.95 (0.88–1.03)	0.220
Child sex						
Female	4992	36.5	1.00 (ref)		1.00 (ref)	
Male	5503	40.6	1.11 (1.05–1.19)	<0.001	1.15 (1.08–1.22)	<0.001
Child age group						
48–59 months	2913	36.2	1.00 (ref)		1.00 (ref)	
36–47 months	2916	38.6	1.07 (0.98–1.17)	0.154	1.08 (1.00–1.17)	0.059
24–35 months	2684	38.6	1.07 (0.97–1.17)	0.165	1.11 (1.02–1.20)	0.012
12–23 months	1982	42.5	1.17 (1.07–1.28)	<0.001	1.18 (1.08–1.28)	<0.001
Maternal education						
Higher	2014	32.9	1.00 (ref)		1.00 (ref)	
Secondary	5716	37.7	1.15 (1.05–1.25)	0.002	1.21 (1.11–1.32)	<0.001
Primary	2027	44.5	1.35 (1.22–1.50)	<0.001	1.42 (1.29–1.57)	<0.001
Pre-primary/none	738	44.6	1.35 (1.19–1.54)	<0.001	1.47 (1.30–1.66)	<0.001
Residence						
Rural	7886	33.9	1.00 (ref)		1.00 (ref)	
Urban	2609	50.1	1.48 (1.38–1.59)	<0.001	1.08 (1.00–1.17)	0.045

Division						
Barishal	1079	29.0	1.00 (ref)		1.00 (ref)	
Chattogram	2102	42.5	1.47 (1.26–1.71)	<0.001	1.39 (1.19–1.62)	<0.001
Dhaka	1765	65.8	2.27 (1.97–2.61)	<0.001	1.82 (1.57–2.12)	<0.001
Khulna	1595	18.0	0.62 (0.51–0.75)	<0.001	0.63 (0.52–0.77)	<0.001
Mymensingh	644	31.0	1.07 (0.86–1.33)	0.554	1.01 (0.81–1.25)	0.946
Rajshahi	1135	16.0	0.55 (0.44–0.69)	<0.001	0.56 (0.44–0.70)	<0.001
Rangpur	1505	19.6	0.68 (0.56–0.81)	<0.001	0.70 (0.58–0.84)	<0.001
Sylhet	670	47.5	1.64 (1.39–1.94)	<0.001	1.59 (1.34–1.89)	<0.001
Wealth quintile						
Poorest	2793	30.3	1.00 (ref)		1.00 (ref)	
Poorer	2476	30.7	1.01 (0.91–1.13)	0.806	1.02 (0.92–1.14)	0.672
Middle	2117	36.8	1.21 (1.10–1.34)	<0.001	1.10 (0.99–1.22)	0.071
Richer	1835	47.3	1.56 (1.42–1.72)	<0.001	1.09 (0.98–1.22)	0.125
Richest	1274	52.1	1.72 (1.55–1.90)	<0.001	1.07 (0.93–1.23)	0.348

PR = prevalence ratio from modified Poisson regression. † Survey-weighted percentage of children with elevated BLLs (≥5 μg/dL) in each category. ‡ Adjusted PR from a single model that included all listed factors simultaneously (n = 10,277 children with complete data on all factors, of the 10,495 in the primary analytic sample). n is the unweighted number of children in each category among the 10,495 children of the primary analytic sample; rows labelled "Missing" are children without data on that factor. Crude PRs used all children with data on the factor concerned (soil lead, n = 10,411; occupational contact, n = 10,433; pica, n = 10,493; cooking fuel, n = 10,494; neighbourhood industry, n = 10,400; all other factors, n = 10,495). Household soil lead was measured directly from a household soil sample; modelled continuously as ln(soil lead), the adjusted PR per 1-SD increment was 1.08 (95% CI 1.05–1.10, p<0.001). The smaller sample of the adjusted model reflects missing data on the environmental and behavioural variables specific to this table (soil lead, pica frequency, neighbourhood industry proximity), which were not required for the primary growth analyses (N = 10,495). Estimates are from survey-weighted models (PSU, stratum, blood-specific weight). Soil-lead quartiles were defined from the survey-weighted distribution (blood-specific weight), so the four quartile groups differ in size.

Table S10. Associations of elevated BLLs (≥5 µg/dL) with anthropometric outcomes.

Outcome	Model 1<br>Adjusted β (95% CI)	P value	Model 2<br>Adjusted β (95% CI)	P value
HAZ	−0.10 (−0.17, −0.04)	0.001	−0.11 (−0.17, −0.05)	0.001
WAZ	−0.07 (−0.13, −0.01)	0.015	−0.07 (−0.13, −0.01)	0.017
WHZ	−0.02 (−0.08, 0.04)	0.536	−0.02 (−0.07, 0.04)	0.612

Values were adjusted β coefficients and 95% confidence intervals from linear regression models. β represents the adjusted mean difference in the outcome comparing children with BLLs ≥5 µg/dL with those with concentrations <5 µg/dL. HAZ = height-for-age z-score; WAZ = weight-for-age z-score; WHZ = weight-for-height z-score; CI = confidence interval; SD = standard deviation.

Model 1 included elevated BLLs as exposure and was adjusted for covariates (child sex, child age, maternal education, place of residence, division, and household wealth).

Model 2 is a mutually metal adjusted model including elevated BLLs as exposure, and arsenic, cadmium, and mercury as co-occurring exposures (as continuous natural log-transformed concentrations standardized to 1 SD), and adjusting for covariates (child sex, child age, maternal education, place of residence, division, and household wealth).

Table S11. Associations of elevated BLLs (≥5 µg/dL) with binary anthropometric outcomes.

Outcome	Model 1:<br>Adjusted PR (95% CI)	P value	Model 2:<br>Adjusted PR (95% CI)	P value
**Stunting**	1.13 (1.04, 1.23)	0.003	1.14 (1.05, 1.23)	0.003
**Wasting**	1.08 (0.94, 1.23)	0.270	1.08 (0.94, 1.24)	0.266
**Underweight**	1.11 (1.01, 1.21)	0.023	1.10 (1.01, 1.20)	0.032
**CIAF**	1.10 (1.03, 1.17)	0.004	1.10 (1.03, 1.18)	0.004

Values were adjusted prevalence ratios (PRs) and 95% confidence intervals from modified Poisson regression models. PRs compare children with BLLs ≥5 µg/dL with those with concentrations <5 µg/dL.

Model 1 included elevated BLLs as exposure and was adjusted for covariates (child sex, child age, maternal education, place of residence, division, and household wealth).

Model 2 is a mutually metal adjusted model including elevated BLLs as exposure, and arsenic, cadmium, and mercury as co-occurring exposures (as continuous natural log-transformed concentrations standardized to 1 SD), and adjusting for covariates (child sex, child age, maternal education, place of residence, division, and household wealth).

Table S12. Associations of elevated BLLs (≥5 µg/dL) with specific patterns of composite anthropometric failure.

CIAF category vs. no failure[a]	Model 1: Adjusted PR (95% CI)	P value	Model 2: Adjusted PR (95% CI)	P value
Wasting only	0.93 (0.67–1.29)	0.652	0.92 (0.66–1.30)	0.647
Wasting + underweight	1.24 (1.02–1.51)	0.031	1.24 (1.02–1.51)	0.032
Stunting + wasting + underweight	1.17 (0.94–1.45)	0.168	1.17 (0.94–1.46)	0.165
Stunting + underweight	1.17 (1.02–1.33)	0.024	1.16 (1.01–1.33)	0.035
Stunting only	1.22 (1.05–1.42)	0.010	1.24 (1.06–1.44)	0.006
Underweight only	1.00 (0.74–1.35)	0.978	0.97 (0.71–1.34)	0.869

Values were adjusted prevalence ratios (PRs) and 95% confidence intervals from modified Poisson regression models. PRs compare children with BLLs ≥5 µg/dL with those with concentrations <5 µg/dL. PR = prevalence ratio; CI = confidence interval; CIAF = composite index of anthropometric failure.

[a] Separate modified Poisson regression model was fitted for each of the six CIAF categories, with children no anthropometric failure as the reference category.

Model 1 included elevated BLLs as exposure and was adjusted for covariates (child sex, child age, maternal education, place of residence, division, and household wealth).

Model 2 is a mutually metal adjusted model including elevated BLLs as exposure, and arsenic, cadmium, and mercury as co-occurring exposures, and adjusting for covariates (child sex, child age, maternal education, place of residence, division, and household wealth).

Table S13. Sensitivity analysis of the association between solid-fuel cooking and elevated blood lead levels (BLLs ≥5 µg/dL): stepwise adjustment for socioeconomic and geographic factors, Bangladeshi children aged 12–59 months (N=10,277).

Adjustment step (cumulative)	Solid-fuel vs clean-fuel cooking, APR (95% CI)	p-value
Crude (solid-fuel cooking only)	0.52 (0.49–0.55)	<0.001
+ child sex and age group	0.52 (0.48–0.55)	<0.001
+ maternal education	0.49 (0.46–0.52)	<0.001

+ urban/rural residence	0.53 (0.49–0.57)	<0.001
+ division (8 divisions)	0.73 (0.68–0.78)	<0.001
+ household wealth quintile	0.75 (0.69–0.81)	<0.001
Full final model, i.e. + soil lead quartile, household occupational lead contact, pica, improved sanitation, paint condition, neighbourhood industry (as in Table S9)	0.78 (0.72–0.85)	<0.001
Full final model with district (67 reporting domains) in place of division	0.91 (0.83–0.99)	0.029

Values were adjusted prevalence ratios (APRs) and 95% confidence intervals for solid-fuel versus clean-fuel cooking as the exposure and elevated BLLs (≥5 µg/dL) as the outcome, from modified Poisson regression with survey weights (blood-specific weight, PSU, stratum). Because solid-fuel cooking was inversely associated with elevated BLLs in the main determinants analysis (Table S9), the association was re-estimated with covariate blocks added cumulatively (child sex and age group, maternal education, residence, division and household wealth), and all models were fitted to the same 10,277 children with complete data on every variable in the final model (8,684 in solid-fuel and 1,593 in clean-fuel households). The full final model includes all factors in Table S9; the last row replaces division with the 67 reporting domains (64 districts plus Dhaka North, Dhaka South and Chattogram City Corporations). APR = adjusted prevalence ratio; BLLs = blood lead levels; CI = confidence interval.

Table S14. Weighted prevalence of solid-fuel cooking and of elevated BLLs by division, Bangladeshi children aged 12–59 months (children with non-missing cooking-fuel data, N=10,494).

Division	n	Solid-fuel cooking, weighted % (95% CI)	Elevated BLL (≥5 µg/dL), weighted % (95% CI)
Rajshahi	1,135	89.8 (87.6–92.0)	16.0 (13.1–18.8)
Khulna	1,595	92.8 (91.1–94.4)	18.0 (15.5–20.5)
Rangpur	1,505	93.7 (92.1–95.4)	19.6 (17.1–22.1)
Barishal	1,079	92.5 (90.0–94.9)	29.0 (25.1–32.9)
Mymensingh	644	88.0 (83.9–92.1)	31.0 (25.6–36.3)
Chattogram	2,102	73.9 (71.2–76.7)	42.5 (39.3–45.8)
Sylhet	670	84.7 (80.6–88.8)	47.5 (42.7–52.2)
Dhaka	1,764	51.0 (47.9–54.1)	65.8 (63.1–68.5)

n = unweighted number of children with non-missing cooking-fuel data. Values are survey-weighted percentages with design-based 95% confidence intervals (blood-specific weight, PSU, stratum). These tabulations were used to examine whether solid-fuel use and elevated BLLs differed between divisions. Divisions are ordered by elevated-BLLs prevalence (lowest to highest). BLLs = blood lead levels; CI = confidence interval.

Table S15. Missing data for the analysis variables among children aged 12–59 months, Bangladesh MICS7.

Variable	Children, N	Missing, n (%)
Blood lead, arsenic, cadmium and mercury	17,132	6,498 (37.9)
Height-for-age, weight-for-age and weight-for-height z-scores (HAZ, WAZ, WHZ) and CIAF	18,913	941 (5.0)
Maternal education	18,913	4 (0.02)
Child age	18,913	8 (0.04)
Child sex, residence, division, household wealth index	18,913	0 (0.0)

Blood metals were assessed among the 17,132 children eligible for blood collection; all other variables among the 18,913 children aged 12–59 months, which includes eight children with missing age who were eligible for blood collection. Anthropometry was classed as missing if any WHO z-score was missing or flagged as implausible. CIAF = composite index of anthropometric failure.

Table S16. Sociodemographic and anthropometric comparison of age-eligible children (12–59 months) with and without a valid blood lead result, Bangladesh MICS7.

Characteristic	Category	Valid blood lead result (n=10,634), n (%)	No valid blood lead result (n=6,498), n (%)	P value
Child sex	Male	5,578 (52.5)	3,307 (51.3)	0.177
	Female	5,056 (47.5)	3,191 (48.7)	
Child age group (months)	12-23	2,024 (19.2)	2,035 (31.5)	<0.001
	24-35	2,727 (26.0)	1,706 (26.2)	
	36-47	2,937 (26.9)	1,507 (23.6)	
	48-59	2,946 (27.9)	1,242 (18.7)	
Maternal education	Pre-primary/none	750 (7.0)	402 (5.7)	<0.001
	Primary	2,051 (19.2)	1,112 (16.7)	
	Secondary	5,785 (54.7)	3,314 (51.4)	
	Higher	2,045 (19.1)	1,669 (26.2)	
Place of residence	Urban	2,643 (27.0)	2,000 (32.7)	<0.001

	Rural	7,991 (73.0)	4,498 (67.3)	
Division	Barishal	1,089 (6.6)	355 (3.3)	<0.001
	Chattogram	2,122 (20.9)	1,570 (25.8)	
	Dhaka	1,793 (20.4)	1,823 (30.2)	
	Khulna	1,619 (11.9)	723 (8.7)	
	Mymensingh	658 (8.5)	401 (8.2)	
	Rajshahi	1,148 (12.7)	579 (9.9)	
	Rangpur	1,525 (13.1)	601 (7.8)	
	Sylhet	680 (5.9)	446 (6.1)	
Household wealth index	Poorest	2,825 (23.6)	1,269 (16.4)	<0.001
	Poorer	2,509 (22.2)	1,186 (16.7)	
	Middle	2,149 (20.3)	1,252 (19.0)	
	Richer	1,860 (19.1)	1,306 (22.4)	
	Richest	1,291 (14.8)	1,477 (25.5)	
Stunting	Yes	2,722 (25.9)	1,446 (25.1)	0.379
Wasting	Yes	1,496 (14.1)	654 (11.1)	<0.001
Underweight	Yes	2,803 (26.7)	1,225 (20.8)	<0.001
CIAF (any failure)	Yes	4,046 (38.3)	2,028 (35.0)	<0.001

n is the unweighted number of children among the 17,132 children aged 12–59 months who were eligible for blood collection; the group without a valid result includes children whose blood sample was not collected and 27 children with an invalid laboratory result. Percentages are survey-weighted column percentages using the general child weight, because the blood-specific weight is available only for children with a blood result. P values are from Rao–Scott chi-square tests. Stunting, wasting, underweight and CIAF are based on WHO growth-standard z-scores among children with valid anthropometry. Children with missing maternal education (n=4) are not shown.
